# Establishment and evaluation of glucose-modified nanocomposite liposomes for the treatment of cerebral malaria

**DOI:** 10.1186/s12951-022-01493-8

**Published:** 2022-07-06

**Authors:** Ya Tian, Zhongyuan Zheng, Xi Wang, Shuzhi Liu, Liwei Gu, Jing Mu, Xiaojun Zheng, Yujie Li, Shuo Shen

**Affiliations:** 1 Institute of Chinese Materia Medica, China Academy of Chinese Medical Sciences, Beijing, 100700 People’s Republic of China; 2 The Hospital of Nanbu County, Sichuan, People’s Republic of China; 3 Chinese Traditional Medicine Resource Center, China Academy of Chinese Medical Sciences, Beijing, 100700 People’s Republic of China; 4 Pharmacy Department of the first hospital of Shanxi Medical University, Shanxi, 10114 People’s Republic of China

**Keywords:** Cerebral malaria, Blood Brain Barrier, Brain-targeted liposomes, GLUT1, Molecular dynamics

## Abstract

Cerebral malaria (CM) is a life-threatening neurological complication caused by Plasmodium falciparum. About 627,000 patients died of malaria in 2020. Currently, artemisinin and its derivatives are the front-line drugs used for the treatment of cerebral malaria. However, they cannot target the brain, which decreases their effectiveness. Therefore, increasing their ability to target the brain by the nano-delivery system with brain-targeted materials is of great significance for enhancing the effects of antimalarials and reducing CM mortality. This study used glucose transporter 1 (GLUT1) on the blood–brain barrier as a target for a synthesized cholesterol-undecanoic acid–glucose conjugate. The molecular dynamics simulation found that the structural fragment of glucose in the conjugate faced the outside the phospholipid bilayers, which was conducive to the recognition of brain-targeted liposomes by GLUT1. The fluorescence intensity of the brain-targeted liposomes (na-ATS/TMP@lipoBX) in the mouse brain was significantly higher than that of the non-targeted liposomes (na-ATS/TMP@lipo) in vivo (P < 0.001) after intranasal administration. The infection and recurrence rate of the mice receiving na-ATS/TMP@lipoBX treatment were significantly decreased, which had more advantages than those of other administration groups. The analysis of pharmacokinetic data showed that na-ATS/TMP@lipoBX could enter the brain in both systemic circulation and nasal-brain pathway to treat malaria. Taken together, these results in this study provide a new approach to the treatment of cerebral malaria.

**Graphical Abstract:**

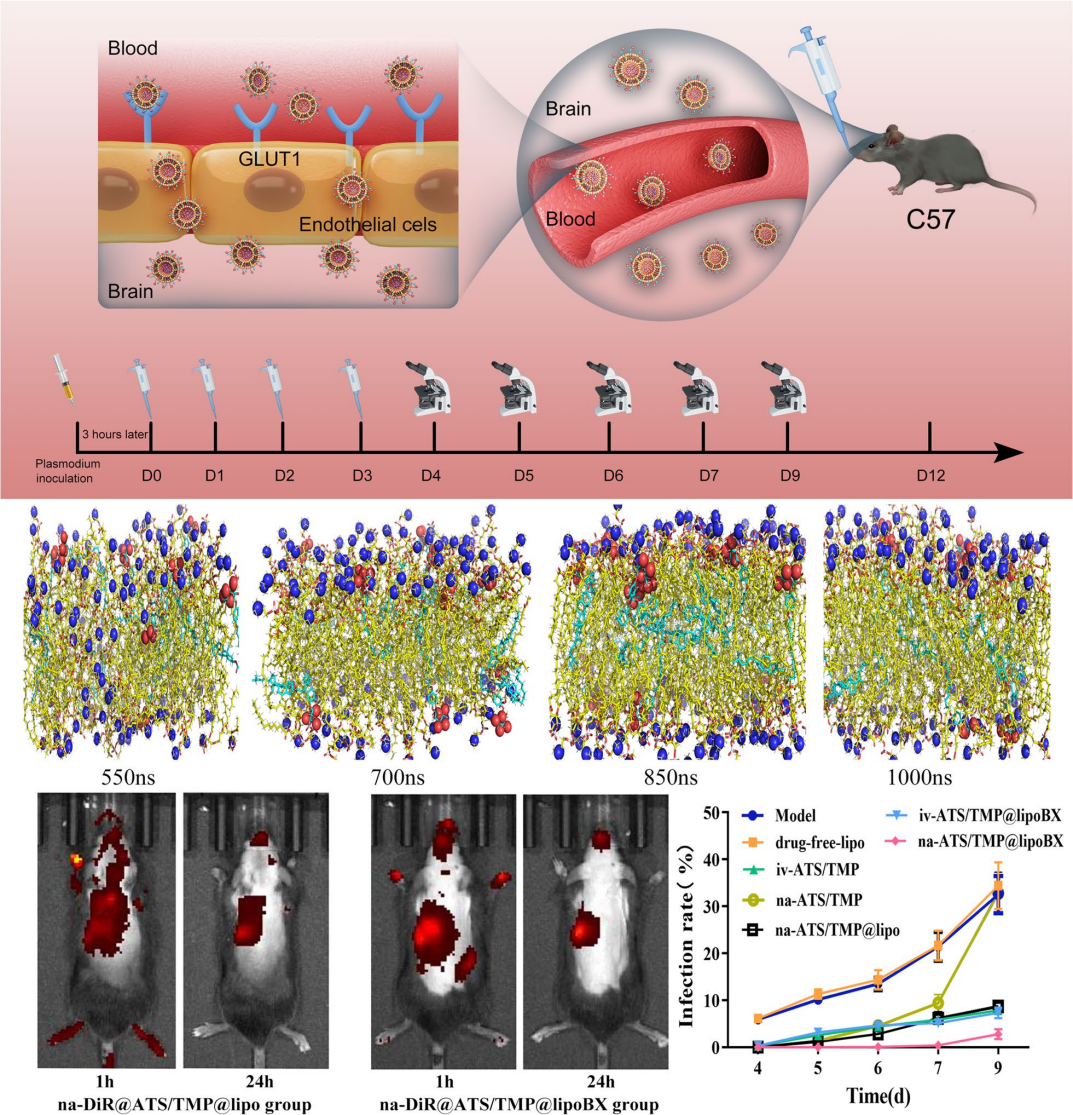

**Supplementary Information:**

The online version contains supplementary material available at 10.1186/s12951-022-01493-8.

## Introduction

Cerebral malaria (CM) is a life-threatening neurological complication caused by *P. falciparum* that mainly affects children in Africa and adults in Asia [1–3]. According to the WHO World Malaria Report, more than 627,000 patients died of malaria in 2020, among which CM accounted for 90% of the deaths [4, 5]. In addition, one-third of CM survivors suffered from long-term neurological and cognitive deficits, such as abnormal behavior, epilepsy, and impaired motor function [5–7].

Artemisinin extracted from *Artemisia annua* is currently the front-line medication for treating malaria. It is a sesquiterpene lactone drug with a peroxide group and it has characterizations such as high efficiency, quick effect, efficient protozoan killing and low toxicity [8–10]. Artemisinin-based combination therapy (ACT), for which the cure rate is higher than 90%, has been widely used in treating malaria in countries worldwide. It can powerfully and rapidly kill malaria-causing parasites in the intraerythrocytic stage and quickly control clinical symptoms [11–14]. However, artemisinin and its derivatives cannot target the brain in the treatment of CM, which leads to they cannot accumulate in the brain. Moreover, 10–20% of CM patients still die even after being treated with artemisinin [15, 16]. Therefore, it is important to improve the brain-targetability of artemisinin and its derivatives to enhance their anti-cerebral malaria efficacy and decrease the mortality of these patients.

The blood–brain barrier (BBB) is an inevitable obstacle during the treatment of brain diseases. It is the barrier between the blood and the cerebrospinal fluid, and while it prevents harmful materials from entering the brain, it also poses a challenge to the treatment of brain diseases [17, 18]. Fortunately, many endogenous substance transporters in the BBB, such as glucose transporter 1 (GLUT1), can efficiently transport glucose into the brain [19–21]. Many studies have shown that directly modifying glucose with drugs or nanoparticles can effectively transport drugs into the brain [22, 23]. In recent years, a large number of nano-preparations have been developed that can cross the blood–brain barrier, such as inorganic nanoparticles [24–26], phospholipid nanoparticles [27–29], nanogels [30, 31] and polymers [32–34]. Among these nanoparticles, liposomes have received extensive attention and have been the topics of research due to their mature preparation technology, high membrane permeability and easy modification, and many of these drugs have been applied in the clinic [35–37]. Another way to improve the brain-targetability of drugs is intranasal administration. This modality can be used to bypass the blood–brain barrier and allow drugs to enter the central nervous system directly, which has the advantages of good brain targetability, high bioavailability, avoidance of the first-pass effect of the liver, convenient use and rapid absorption [38–40].

**Fig. 1:**
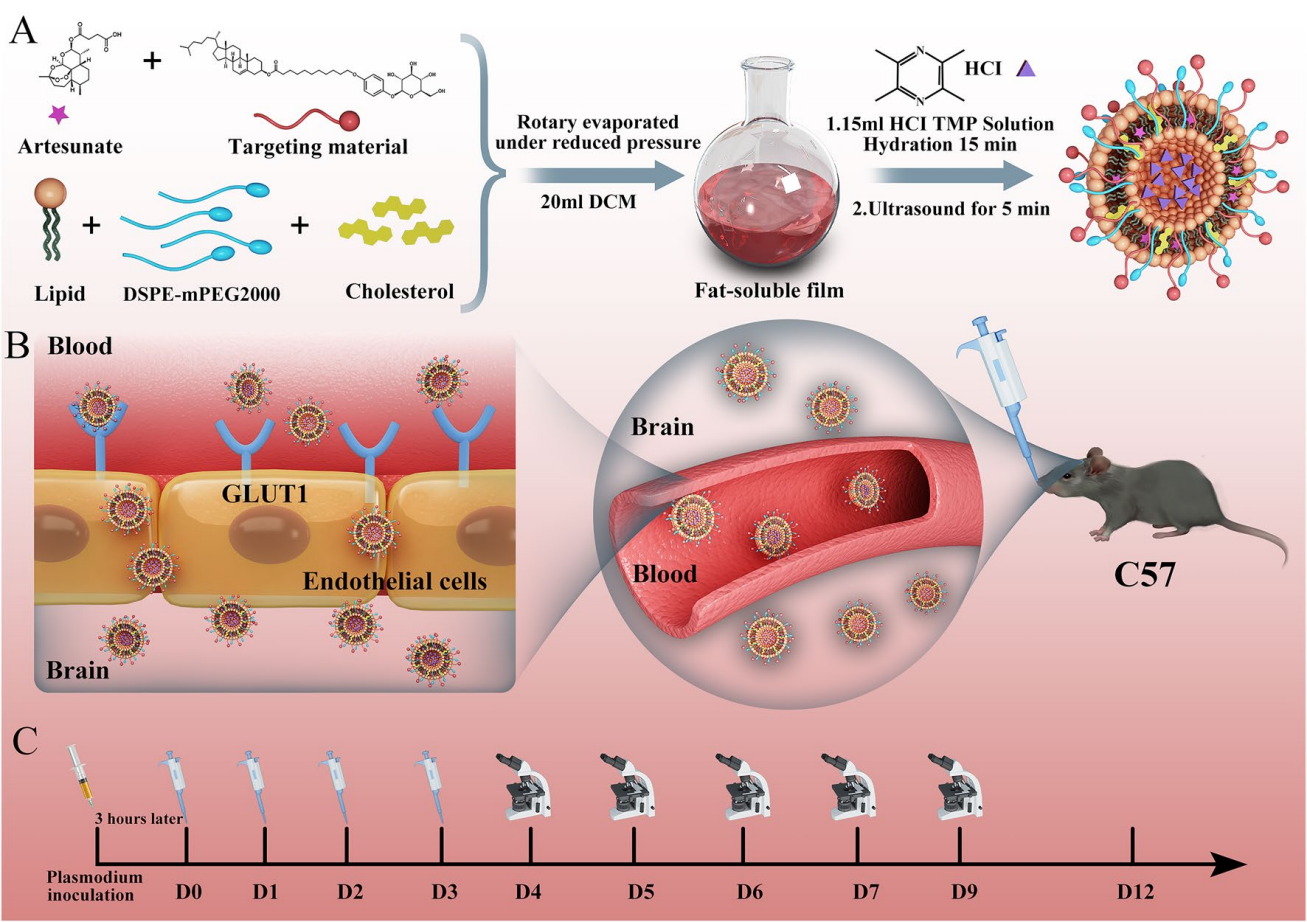
Preparation and evaluation of brain-targeted antimalarial liposomes. **A** The preparation method of brain-targeted liposomes. **B** The process of brain-targeted liposomes crossing the blood–brain barrier. **C** Pearson’s 4-day suppression test was used to evaluate the antimalarial efficacy of brain-targeted liposomes

In this study, glucose transporter 1 (GLUT1) at the blood–brain barrier was used as a target for the design of targeted materials and the preparation of brain-targeted liposomes. Through molecular dynamics simulation, the feasibility of each targeted material and phospholipid to form a bilayer structure was evaluated to screen suitable GLUT1 targeted materials for the preparation of brain-targeted liposomes. Furthermore, a preferred targeting conjugate (cholesterol-undecanoate-glucose conjugate) screened from molecular dynamics simulations was synthesized for further study. The liposomes were prepared by the thin-film dispersion method, and then the characteristics of its particle size, encapsulation efficiency and drugs released in vitro etc. were evaluated. In our previous study, artesunate (ATS) combined with representative components of herbs (typically used in Chinese medicine to treat brain diseases), including *Salvia miltiorrhiza* and *chuanxiong*, were tested to determine whether their effects were synergistic. It was found that ATS combined with ligustrazine hydrochloride (TMP) in *chuanxiong* can significantly improve the efficacy of anticerebral malaria in a mouse model [7, 41, 42]. Therefore, in this study, ATS and TMP were co-encapsulated into liposomes to evaluate their anticerebral malaria efficacy and brain targetability (Fig. 1). Finally, the pharmacokinetics study of brain-targeted liposomes was also performed to measure the concentration of ATS and TMP in both plasma and the brain after intravenous administration (i.v.) and intranasal administration (i.n.), respectively.

## Materials and methods

### Materials

Arbutin, Tetraethylammonium bromide (TBAB), 1-(3-Dimethylaminopropyl)-3-ethylcarbodiimide hydrochloride (EDCI), 4-dimethylaminopyridine (DMAP), 11-bromoundecanoic acid and cholesterol were purchased from Adamas Reagent (China) Company (Shanghai, China). Anhydrous potassium carbonate, Dichloromethane, Methanol, Petroleum ether, Ethyl acetate and dimethylformamide were bought from Shanghai Titan Chemical Co., Ltd (Shanghai, China). Ligustrazine Hydrochloride (TMP) was purchased from Hubei Xing Yinhe Chemical Co., Ltd. Artesunate (ATS) was purchased from Kunming Pharmaceutical Group Chongqing Wulingshan Pharmaceutical Group (Chongqing, China). Soy lecithin was purchased from Shanghai Taiwei Pharmaceutical Co., Ltd (Shanghai, China). DSPE-mPEG2000 was purchased from A.V.T. (Shanghai) Pharmaceutical Co., Ltd (Shanghai, China). ATS injection was got from Guilin Pharmaceutical Co., Ltd (Guilin, China). TMP Injection was obtained from Beijing Yongkang Pharmaceutical Co., Ltd (Beijing, China). HPLC column (Phenomenex Luna C18(2), 4.6 × 100 mm, 3 μm) was purchased from Phenomenex (California, USA). Acetonitrile HPLC grade was purchased from Tianjin Concord Technology Co., LTD (Tianjin, China). Centrifugal ultrafiltration tubes were purchased from Merk Millipore Ltd (Darmstadt, Germany). LC–MS (Exion LC-20AC, SCIEX Triple Quad 6500 + mass spectrometer, AB SCIEX, USA).

### Malaria parasite and animals

Plasmodium Pb ANKA strain was obtained from the Fourth Military Medical University (Xian, China). C57BL/6 male mice (15–20 g) were obtained from Beijing Weitong Lihua Co., Ltd (Beijing, China). All experimental procedures were executed according to the guidelines for care and use of laboratory animals of the Institute of Chinese Materia Medica, China Academy of Chinese Medical Sciences.

### Screening target materials

**Fig. 2:**
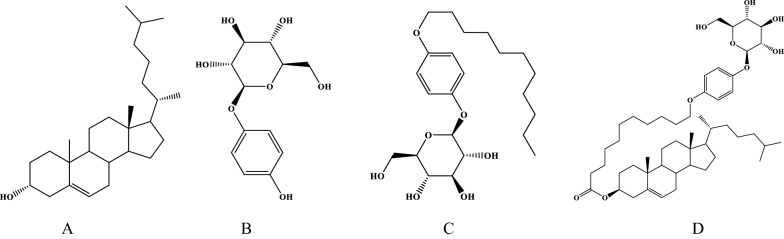
The structure of target materials. **A** cholesterol; **B** arbutin; **C** undecane-glucose conjugate; **D** cholesterol-undecanoate-glucose conjugate

Taking the GLUT1 transporter of the blood–brain barrier as the target, three targeted materials were selected or designed in this study (Fig. 2). Through molecular dynamic simulation, the feasibility of each targeted material and phospholipid to form a bilayer structure was evaluated to screen suitable GLUT1 targeted materials for the preparation of brain-targeted liposomes.

In order to screen the target materials which can stably inserted into the phospholipid bilayers. The self-assembly of soybean lecithin (1-Palmitoyl-2-lauroyl- sn-glycero-3-phosphocholine, PLPC) with cholesterol, arbutin, cholesterol-undecanoate-glucose conjugate and undecane-glucose conjugate were performed, respectively. All simulations were performed using the GROMACS molecular dynamics software suite (version 2020.2) [43], and the GPU acceleration was applied. The topological files of phospholipid, cholesterol, conjugates and arbutin were generated using charmm36 force field [44, 45]. 128 PLPC phospholipids, 13 conjugates, cholesterols, arbutin and 2000 SPC water [46] were added into a cubic box with periodic boundary conditions using gmx insert. The conjugate gradient method was employed to optimize 10,000 steps, and the cutoff value of each step was set to 0.1 nm; then the system was pre-equilibrated at 200 ps, and then the simulation was performed at 1000 ns. In molecular dynamics, the temperature of the system was maintained by independently coupling the lipids, water, and others to an external temperature bath at the appropriate reference temperature with a coupling constant of 0.2 ps using a Berendsen thermostat. The electrostatic interactions were evaluated using particle mesh Ewald (PME) [47]. A cut-off of 10 Å limited the direct space sum and truncated the van der Waals interactions. Bonds involving hydrogen were subjected to length constraints provided by the LINCS algorithm [48]. The Langevin coupling scheme [49], with a collision frequency of 1.0 ps^−1^, was used to regulate temperature while a Berendsen barostat [50] maintained a reference pressure set to 1.0 bar.

### Synthesis of cholesterol-undecanoate-glucose conjugate and its characterization

Based on the results of molecular dynamics simulations, the cholesterol-undecanoate-glucose conjugate was selected for synthesis in this paper. And this material was used to prepare brain-targeted liposomes. 4 g 11 bromo-undecanoic acid, 8.75 g cholesterol (1.5 equiv), 4.32 g EDCI (1.5 equiv) and 552 mg DMAP (0.2 equiv) were added into a 100 ml flask, and 60 ml of dichloromethane was added, then this reaction reacted for overnight at room temperature. After that the solution was directly added to silica gel for further purification. Petroleum ether: Ethyl acetate (100:1, V/V) was used as the eluent. At last, white like powder 3-(11-bromoundecanoate)-cholesterol was obtained (Additional file 1: Fig. S1).

9 g 3-(11-bromoundecanoate)-cholesterol, 5.8 g arbutin (2 equiv), 5.88 g anhydrous potassium carbonate (3 equiv) and 1.4 g TBAB (0.3 equiv) was added to 250 ml round bottom flask, with 80 ml of DMF was added, then put the round-bottomed flask in an oil bath at 95 ℃ heating to reflux for 5 h. Then the solution was poured into 500 ml of pure water to obtain a crude product. Dichloromethane/Methanol (200:1, V/V) was used as an eluent to obtain a white blocky product. Products were determined by ^1^H and ^13^C NMR spectra (Bruker 600 M NMR, Germany), mass spectrometer (Bruker ultraextreme MALDI, Bruker, Germany) and infrared spectrometer (Nicolet iS10 FT-IR, Thermo Fisher Scientific, USA).

### Preparation of ATS/TMP liposomes

#### ATS/TMP-free liposomes for injection administration (drug-free lipo)

300 mg phospholipids, 25 mg cholesterol and 25 mg DSPE-mPEG 2000 were dissolved in 20 ml dichloromethane in a round-bottomed flask; then the dichloromethane was completely removed by rotary evaporation under reduced pressure, after that 15 ml of distilled water was added into the round-bottomed flask and hydrated for 15 min. Finally, probe sonication was performed for 5 min, with a 2/2 s on/off working cycle and output of 260 w power to generate Drug-free lipo.

#### Brain-targeted liposomes for injection administration (iv-ATS/TMP@lipoBX)

300 mg phospholipids, 25 mg cholesterol, 25 mg DSPE-mPEG2000, 25 mg cholesterol-undecanoate-glucose conjugate and 23.4 mg ATS were dissolved in 20 ml dichloromethane in a round-bottomed flask; then the dichloromethane was completely removed by rotary evaporation under reduced pressure, after that 15 ml of distilled water with 31.2 mg TMP was added into the round-bottomed flask and hydrated for 15 min. Finally, probe sonication was performed for 5 min, with a 2/2 s on/off working cycle and output of 260 w power to prepare iv-ATS/TMP@lipoBX.

#### Brain-targeted liposomes for intranasal administration (na-ATS/TMP@lipoBX)

1.5 g phospholipids, 75 mg cholesterol, 150 mg DSPE-mPEG2000, 150 mg cholesterol-undecanoate-glucose conjugate and 180 mg ATS were dissolved in 30 ml dichloromethane in a round-bottomed flask; then the dichloromethane was completely removed by rotary evaporation under reduced pressure, after that 15 ml of distilled water with 240 mg TMP was added into the round-bottomed flask and hydrated for 30 min. Finally, probe sonication was performed for 5 min, with a 2/2 s on/off working cycle and output of 260 w power to prepare na-ATS/TMP@lipoBX.

#### Non-targeted liposomes for intranasal administration (na-ATS/TMP@lipo)

1.5 g phospholipids, 75 mg cholesterol, 150 mg DSPE-mPEG2000 and 180 mg ATS were dissolved in 30 ml dichloromethane in a round-bottomed flask; then the dichloromethane was completely removed by rotary evaporation under reduced pressure, after that 15 ml of distilled water with 240 mg TMP was added into the round-bottomed flask and hydrated for 30 min. Finally, probe sonication was performed for 5 min, with a 2/2 s on/off working cycle and output of 260 w power to prepare na-ATS/TMP@lipo [51, 52].

#### Fluorescent labeled liposomes for intranasal administration

##### Fluorescent labeled brain-targeted liposomes for intranasal administration (na-DiR@ATS/TMP@lipoBX)

The absolute ethanol solution of the DiR iodide (DiR) fluorescent probe was added to the dichloromethane solution of the excipients. Next, the preparation of na-DiR@ATS/TMP@lipoBX was completed according to the subsequent preparation process of na-ATS/TMP@lipoBX.

##### Fluorescent labeled non-targeted liposomes for intranasal administration (na-DiR@ATS/TMP@lipo)

The absolute ethanol solution of the DiR fluorescent probe was added to the dichloromethane solution of the excipients. Next, the preparation of na-DiR@ATS/TMP@lipo was completed according to the subsequent preparation process of na-ATS/TMP@lipo.

### Identification of characterizations of liposomes

#### Particle size

Malvern laser particle size analyzer (Malvern ZS 90, Malvern Instruments, UK.) was used to investigate the particle size of liposomes. 1 ml liposomes were added into the container and the measurement was carried out in triplicate after equilibrating at 25 °C for 30 s. The results were the mean values.

#### Transmission electron microscopy (TEM)

1 ml liposomes were diluted using drilled water to 2 ml, and the diluted liposomes were dropped on 200-mesh copper grids. Transmission electron microscopy (TEM) (H-7650 Transmission Electron Microscope, Hitachi, Japan) was used to observe the structure of liposomes after they naturally dried.

#### Differential scanning calorimetry (DSC)

The calorimetric experiments were carried out in a microcalorimeter (Nano DSC, TA, USA). The sample was scanned from 25 ℃ to 95 ℃, heating rate of 1 ℃ per minute, sample chamber pressure was 3 atmospheres.

### Determination of liposomes encapsulation efficiency and in vitro release

#### Method for determining the HPLC contents of ATS and TMP

##### ATS

HPLC conditions were as follows. Chromatographic column: C18 (2) 100 Å column (3 μm, 4.6 × 100 mm, Phenomenex); mobile phase: acetonitrile/water containing phosphate buffer solution of 1 ml/L (50:50, v/v); flow rate: 1.0 ml/min; column temperature: 30 ℃; detection wavelength: 216 nm; injection volume: 20 μl. The contents were determined by Waters HPLC (e2695-2998 HPLC, Waters, USA).

##### TMP

HPLC conditions were as follows. Chromatographic column: C18 (2) 100 Å column (3 μm, 4.6 × 100 mm, Phenomenex); mobile phase: methanol/water (45:55, v/v); flow rate: 0.8 ml/min; column temperature: 30 °C; detection wavelength: 295 nm; injection volume: 20 μl. The contents were determined by Waters HPLC (e2695-2998 HPLC, Waters, USA).

##### Determination of liposome encapsulation efficiency

To identify the encapsulation efficiency (EE) of ATS and TMP in liposomes, the liposomes were centrifuged at 5000 g for 90 min to remove the unencapsulated drugs. The amounts of ATS and TMP in the supernatant were measured by HPLC with the above conditions. The EE (%) values of ATS and TMP in liposomes were denoted as the ratio between the amount of encapsulated drug and total drug amount in the formulation (n = 3) [51]:

$$\mathrm{EE} \left(\%\right)= \frac{{C}_{total}-{C}_{free}}{{C}_{total}} \times 100\%$$

where “C_total_” was the weight of total ATS or TMP in liposomes and“C_free_” was the mass of the free drug from the liposomes.

#### In vitro drugs release liposomes

In vitro drug-release of ATS and TMP from na-ATS/TMP@lipoBX and iv-ATS/TMP@lipoBX were studied using the dialysis method, respectively. 1 ml na-ATS/TMP@lipoBX liposome and 2 ml iv-ATS/TMP@lipoBX liposome were placed in dialysis bags (MWCO 7 kDa), respectively. The dialysis bags were placed in normal saline release solution containing 20% ethanol at 37 ℃ with stirring speed of 75 rpm. At predetermined time intervals, 1 mL of release solution was taken out for content determination and 1 ml fresh release solution was refilled. The number of drugs released was determined by HPLC according to “Method for determining the HPLC contents of ATS and TMP” (n = 3).

#### Stability study of liposomes

##### Placement stability of liposomes

iv-ATS/TMP@lipoBX and na-ATS/TMP@lipoBX were stored at 4 °C after preparation, and the particle size and encapsulation efficiency of the liposomes were measured at 0,1,2,3 and 4 weeks after preparation by the above method “Particle size” and “Determination of liposome encapsulation efficiency”. The RSD of particle size and encapsulation efficiency was calculated to evaluate the placement stability of liposomes.

##### Stability of liposomes in serum

iv-ATS/TMP@lipoBX and na-ATS/TMP@lipoBX were added to the serum of cerebral malaria model mice (C57BL/6) in appropriate proportions and incubated at 37 °C. The particle size of liposomes was measured at 0 h,2 h,4 h,8 h,12 h,24 h and 48 h by the above method “Particle size” respectively, and the RSD of particle size was calculated to evaluate the stability of liposomes in serum.

### In vivo brain-targetability evaluation

The in vivo tissue distributions of drug-loaded liposomes were evaluated by a fluorescence imaging system (IVIS Spectrum, PerkinElmer, USA) on mice. C57BL/6 mice infected with Plasmodium falciparum were depilated on their brains and backs before the experiment. Malaria-infected mice were randomly divided into three groups. The first group of mice was intranasally administered with na-DiR@ATS/TMP@lipoBX (brain-targeted liposomes), the second group of mice was intranasally administered with na-DiR@ATS/TMP@lipo (non-targeted liposomes) and the third group of mice was injected with iv-ATS/TMP@lipoBX (brain-targeted liposomes), respectively. At designated time points following intranasal administration, a part of mice was anesthetized with isoflurane and imaged by IVIS Spectrum, another part of the mice was euthanized and the brains were removed for ex vivo fluorescence imaging. The fluorescence intensity of isolated brains was analyzed to evaluate the brain-targetability of liposomes.

### In vivo antimalarial experiment

#### Resuscitation and passage

The Pb ANKA P. *falciparum* strain frozen in liquid nitrogen was placed in a 37 °C water bath. The blood was immediately inoculated into C57BL/6 mice at a 0.2 ml/mouse dose after it was dissolved. When the infection rate of breeding mice reached 15–30%, collected the blood of breeding mice was into anti-coagulation tube, then injected into offspring C57BL/6 mice at the same dose.

#### P. falciparum vaccination and administration

The C57BL/6 mice were weighed and divided into model group, intranasal administration of ATS and TMP solutions group (na-ATS/TMP), intravenous administration of ATS and TMP solutions group (iv-ATS/TMP), intranasal administration of na-ATS/TMP@lipoBX group (na-ATS/TMP@lipoBX), intranasal administration of na-ATS/TMP@lipo group (na-ATS/TMP@lipo), intravenous administration of iv-ATS/TMP@lipoBX solutions group (iv-ATS/TMP@lipoBX), intravenous administration of drug-free lipo group (drug-free lipo) with 10 mice in each group. The infection rate of offspring C57BL/6 mice reached 15 ~ 30%, intraperitoneal injection of 0.2 ml containing 1 × 10^6^ erythrocytes infected with P. falciparum into all above mice was used to establish the malaria model, which was recorded as D0. The mice in the model group were injected intraperitoneally with 0.2 ml of saline.

The antimalarial efficiency of drugs was evaluated by Pearson’s 4-day suppression test in mice. Before administration, the liposomes were sterilized with a 0.22 μm filter membrane on the ultra-clean bench. The liposomes of drug-free lipo and iv-ATS/TMP@lipoBX were injected through the tail vein according to the weight of the mice. Artesunate and ligustrazine hydrochloride (ATS/TMP) solutions, the liposomes of na-ATS/TMP@lipoBX and na-ATS/TMP@lipo were administered by intranasal drip for mice. The method of intranasal drip: Grab neck of mouse to make its nose up, and used a pipette to drop drug into nostrils of mice at a 45°. The dosage of each administration group was artesunate (ATS) 15.6 mg/kg and tetramethylpyrazine hydrochloride (TMP) 20.8 mg/kg.

### Pharmacokinetic and brain biodistribution studies

#### Drug administration

The C57BL/6 mouse cerebral malaria model was established according to the method of “In vivo antimalarial experiment”. The model mice were randomly divided into the intranasal administration group (na-ATS/TMP@lipoBX) and tail vein injection group (iv-ATS/TMP@lipoBX). The dosage of each administration group was artesunate (ATS) 15.6 mg/kg and tetramethylpyrazine hydrochloride (TMP) 20.8 mg/kg.

#### Samples preparation

##### Plasma samples

Blood samples were collected at 5 min, 10 min, 15 min, 30 min, 45 min, 1 h, 2 h, 4 h, 8 h, 12 h and 24 h after administration. These samples were immediately placed in a tube containing heparin sodium (1000 U/mL). Plasma samples were obtained after centrifugation at 4000 rpm for 10 min and an esterase inhibitor was added to the samples. Subsequently, methanol was added to the plasma in the ratio of 1:2 (plasma-methanol) and centrifuged at 14,000 rpm for 5 min to obtain liquid supernatant for quantitative analysis by LC–MS.

##### Brain tissue samples

Mice were euthanized and the brains were dissected. The brain samples were homogenized by adding 4 times their weight of physiological saline to prepare a tissue homogenate. The brain homogenates were processed according to the processing method for the plasma sample, and LC–MS quantitative analysis was carried out.

##### Quantitative LC-MS/MS analysis of components in plasma and brain

The levels of ATS, TMP and dihydroartemisinin (DHA) in plasma or brain were assayed by LC–MS (Exion LC-20AC, SCIEX Triple Quad 6500 + mass spectrometer, AB SCIEX, USA).

LC conditions were as follows: column: Waters UPLC BEH C18 column (1.7 μm, 2.1 mm × 50 mm); mobile phase: water (0.1% formic acid and 5 mM ammonium formate)-acetonitrile (85:15, v/v), flow rate: 0.3 ml/min; column temperature: 35 ℃. Mass spectrum conditions [53] were as follows: electric spray ion source (ESI); multiple response monitoring (MRM) mode; positive ion scanning mode; air curtain pressure (N_2_) was 40 psi, impact air pressure (N_2_) was 9 psi, atomization gas pressure (N_2_) was 55 psi, auxiliary gas pressure (N_2_) was 55 psi; spray voltage was 5500 V; atomization temperature was 550 ℃. The ion pairs of the analytes were as follows: ATS (m/z: 402.1, 267.4, declustering potential: 20 V), DHA (m/z: 302.4, 267.3, declustering potential: 20 V) and TMP: (m/z: 137.1, 80.1, declustering potential: 74 V).

### Hematoxylin and eosin (H&E) staining

After the antimalarial experiment, the mice were sacrificed by cervical dislocation. The heart, liver, spleen, lung, kidney, and brain were collected and fixed with 10% formalin solution. And then the tissue samples were embedded, sectioned, stained with H&E (hematoxylin–eosin) and slice sealed for microscopic observation to examine the histological changes.

### Statistical analysis

Data were expressed as the mean ± standard deviation (SD). Data analysis and comparison were performed by Student’s t-test and one-way ANOVA using GraphPad Prism 8 software (GraphPad Software Inc., La Jolla, CA, USA). Significant difference was regarded as *p < 0.05, **p < 0.01 and ***p < 0.001.

## Results and discussion

### Screening target materials

In the study of molecular dynamics simulation, the phospholipid bilayer structure, the area per lipid, deuterium order and the electron density in the simulation system were selected as parameters to analyze the reliability of the bilayer structure formed by various materials and phospholipid. Based on the results, the preferred targeted material was used for preparation of brain-targeted liposomes.

#### The formation process of lipid bilayer

**Fig. 3:**
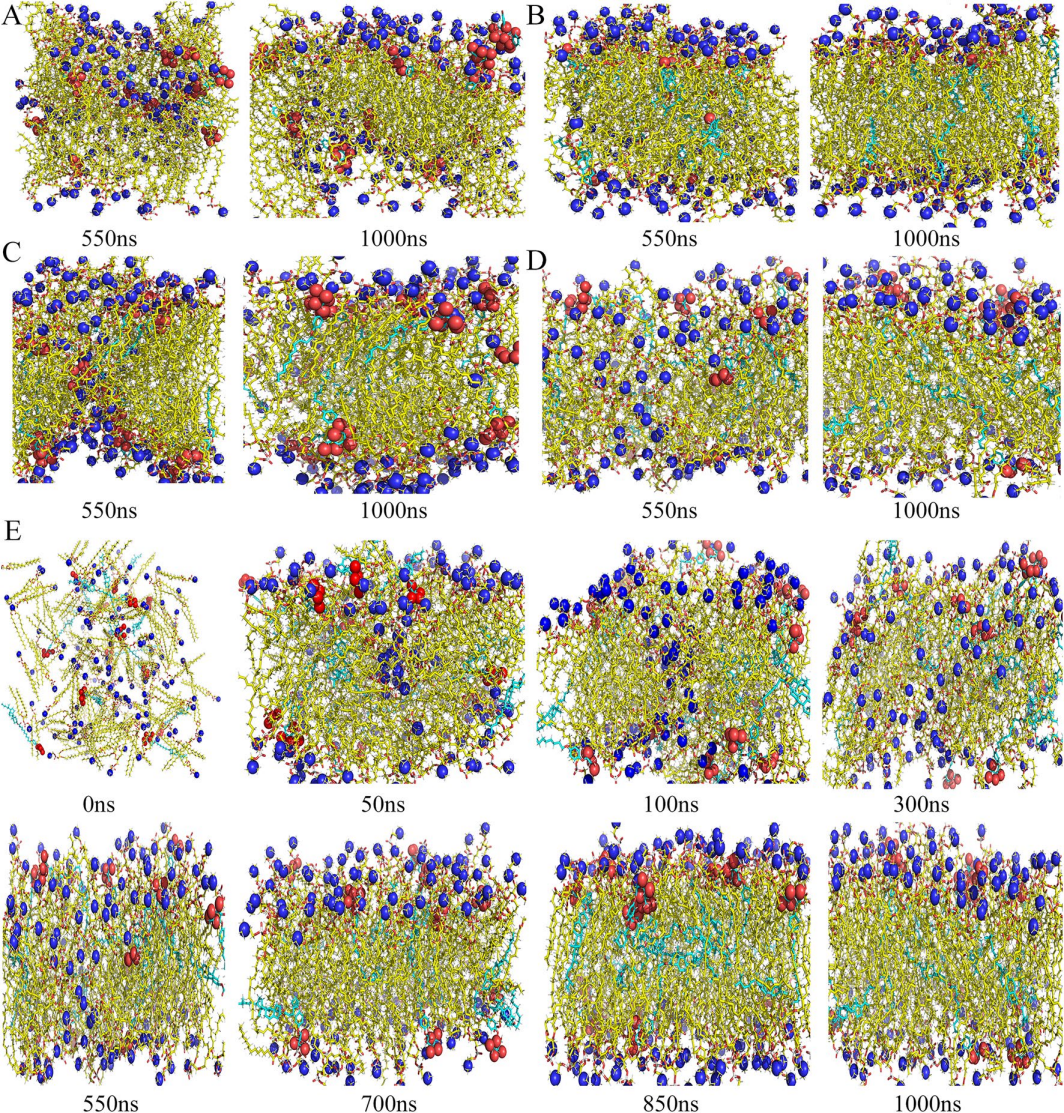
The self-assembly of the phospholipid bilayer at 550 ns and 1000 ns. **A** arbutin.; **B** cholesterol; **C** undecane-glucose conjugate; **D** cholesterol-undecanoate-glucose conjugate. **E** The self-assembly of a lipid bilayer: Snapshots showing the spontaneous self-assembly of PLPC into a bilayer: the starting random mixture (t = 0 ns); a bilayer-like assembly containing a pore (t = 50 ns); an equilibrated bilayer (t = 700 ns). The PLPC and target materials are depicted as yellow and cyan respectively, blue is the nitrogen atom in phospholipids, red is oxygen atom in conjugates. Note that water molecules are not shown

In the study, the molecular dynamic simulation trajectory pictures of phospholipid and materials were taken at 0 ns, 50 ns, 100 ns, 300 ns, 550 ns, 700 ns, 850 ns and 1000 ns. And the morphologies of phospholipid bilayer formed by materials and phospholipid were evaluated intuitively. The results showed that compared with other materials, the cholesterol-undecanoate-glucose conjugate was like cholesterol and could form a stable bilayer structure with phospholipid (Fig. 3A–D and Additional file 1: Figs. S2–S4).

The soybean lecithin and cholesterol-undecanoate-glucose conjugate initially formed a phospholipid bilayer within 50 ns but did not completely form a phospholipid bilayer until 700 ns, and the structure fragments of glucose in the conjugates (red) faced outside the phospholipid bilayer, which was conducive to the recognition of brain targeted liposomes by GLUT1 (Fig. 3E). As mentioned above, the cholesterol-undecanoate-glucose conjugate was selected as target material for further study.

#### The area per lipid at 1000 ns

The area per lipid (A_L_) at 1000 ns was calculated using gridMAT-MD [54]. The A_L_ of the top and bottom layers was 0.65 nm^2^ and 0.69 nm^2^ in the PLPC/cholesterol simulation, respectively. The A_L_ of top and bottom layers in the PLPC/cholesterol-undecanoate-glucose conjugate, PLPC/arbutin and PLPC/undecane-glucose conjugate was 0.43 nm^2^ and 0.42 nm^2^, 0.42nm^2^ and 0.52nm^2^ and 0.52 nm^2^ and 0.48 nm^2^, respectively. It could be found that conjugates (cholesterol-undecanoate-glucose conjugate and undecane-glucose conjugate) and arbutin decreased the A_L_. However, the A_L_ is an inadequate parameter to estimate the quality of a force field in a simulation. It is highly sensitive not only to the force field itself but also to a range of secondary simulation parameters. An alternative to A_L_ as a measure of the quality of a simulation is the volume per lipid (V_L_). V_L_ converges faster than A_L_ and shows less fluctuation than A_L_ [46]. V_L_ can be calculated from the following equation [44, 46]:

$${V}_{L}=\frac{V-{n}_{w}{V}_{w}}{{n}_{L}}$$

where V is the volume of the simulation box and n_L_ and n_w_ are the number of phospholipids (128) and water molecules (2000), respectively. V_w_ is the volume per water molecule. V_w_ = 3.15 × 10^−2^ nm^3^ was found at 323 K and a pressure of 1 bar [46]. The V_L_ was 1.72 nm^3^, 1.77 nm^3^, 1.23 nm^3^ and 1.24 nm^3^ in the PLPC/cholesterol, PLPC/cholesterol-undecanoate-glucose conjugate, PLPC/arbutin and PLPC/undecane-glucose conjugate at 1000 ns, respectively. So, cholesterol-undecanoate-glucose conjugate will not be significantly affected the V_L_ of phospholipid bilayers compared with cholesterol.

#### Deuterium order

**Fig. 4:**
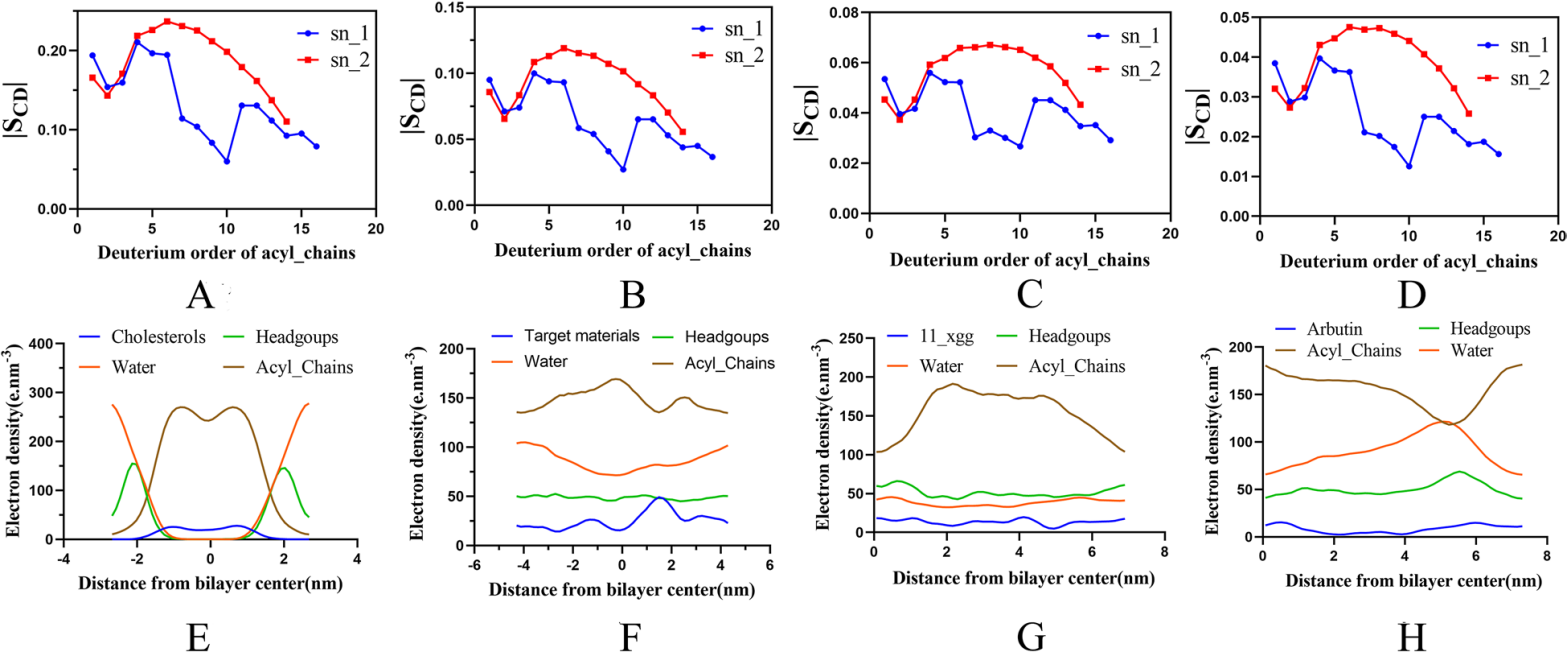
(1) Deuterium order parameter of PLPC/targeted materials. **A** cholesterol; **B** cholesterol-undecanoate-glucose conjugate; **C** undecane-glucose conjugate; **D** arbutin. (2) Electron density profiles of PLPC/targeted materials. **E** cholesterol; **F** cholesterol-undecanoate-glucose conjugate; **G** undecane-glucose conjugate; **H** arbutin

Deuterium order (S_CD_) is a parameter commonly used to characterize the degree of order of fatty chains in phospholipid bilayers. S_CD_ can be calculated from the following equation [46, 55]:

$${S}_{CD}=\frac{1}{2}(3{cos}^{2}{\theta }_{i}-1)$$

where *θ*_*i*_ is the angle between the i^th^ molecular axis and the bilayer normal (z-axis). The deuterium order of the PLPC/cholesterol and PLPC/cholesterol-undecanoate-glucose conjugate, PLPC/undecane-glucose conjugate and PLPC/arbutin are shown in Fig. 4A–D, respectively. The S_CD_ of sn-1 and sn-2 in both simulations exhibited similar changes, but the hydrocarbon chains in the PLPC/conjugate had lower S_CD_. This finding indicates that the S_CD_ of the phospholipids in the conjugate was lower than that of cholesterol. But S_CD_ in cholesterol-undecanoate-glucose conjugate is higher than both arbutin and undecane-glucose conjugate, which means, compare with arbutin and undecane-glucose conjugate, the cholesterol-undecanoate-glucose conjugate can make phospholipid bilayers more order and compact [56, 57]. It could conclude that PLPC/cholesterol-undecanoate-glucose conjugate liposomes could encapsulate more drugs and stably in *vivo* deliver drugs to target the brain.

#### The electron density in the simulation system

The electron density in the simulation system was analyzed, and the electrons in the hydrocarbon chain in PLPC/cholesterol were mainly accumulated in the center of the simulation box. The charges of the water and phospholipid heads were mainly distributed on the edge of the box (Fig. 4E). In the PLPC/ cholesterol-undecanoate-glucose conjugate, PLPC/undecane-glucose conjugate and PLPC/arbutin, the charge distribution was relatively uniform (Fig. 4F–H). In the PLPC/cholesterol-undecanoate-glucose conjugate, the charges of the phospholipid hydrocarbon chains were mainly distributed at − 0.5 Å in the center of the box. The charges of the cholesterol-undecanoate-glucose conjugates were mainly concentrated at 1.8 Å in the center of the box.

In summary, through the analysis of dynamic simulation data, the results showed that the cholesterol-undecanoate-glucose conjugate could better form a stable and ordered phospholipid bilayer structure with phospholipid than other targeted materials. So, the cholesterol-undecanoate-glucose conjugate was selected as GLUT1 targeted material for further study.

### Synthesis of cholesterol-undecanoate-glucose conjugate and its characterization

**Fig. 5:**
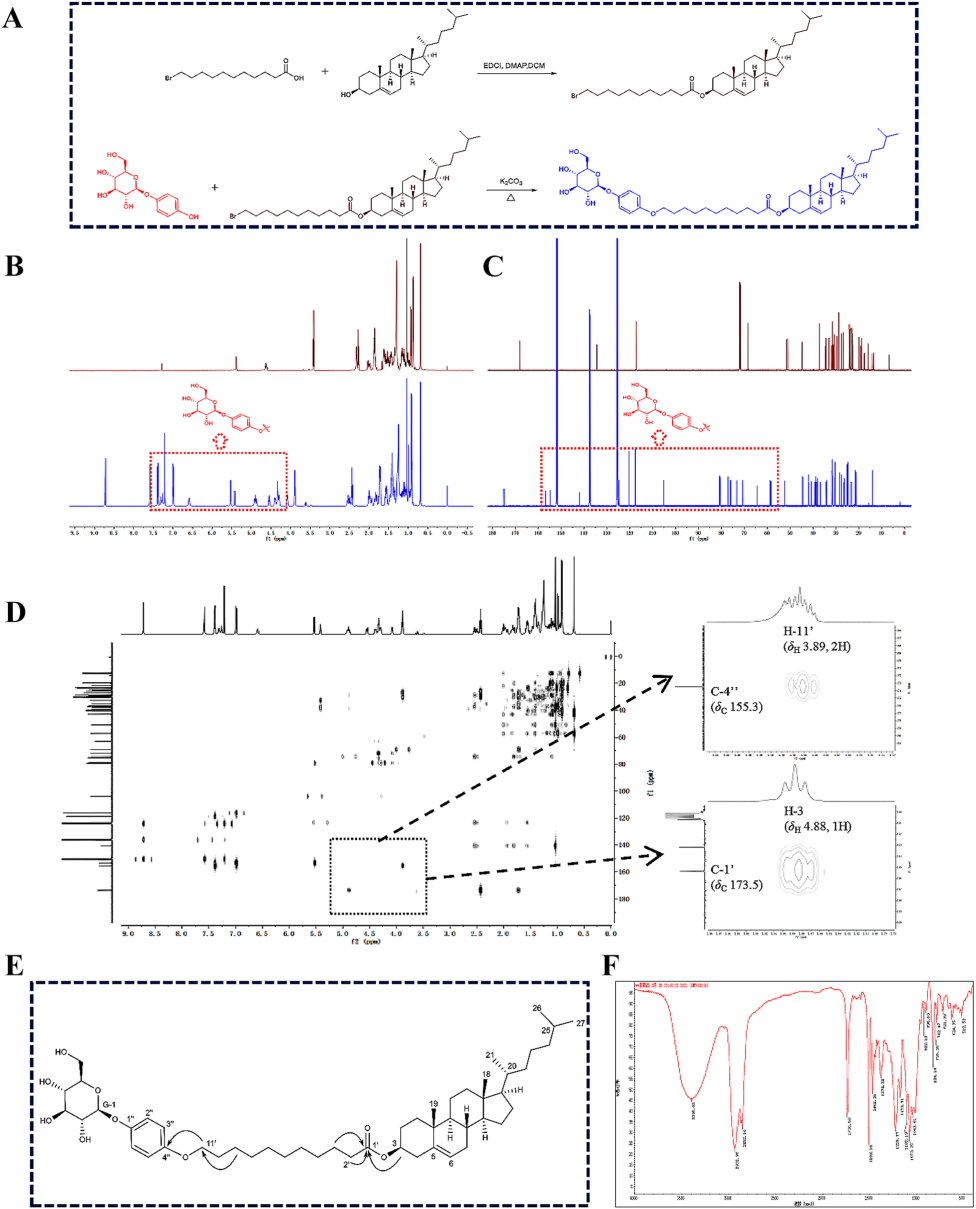
Synthesis of cholesterol-undecanoate-glucose conjugate and analysis of the molecular structure of the conjugate. **A** The synthetic route of cholesterol-undecanoate-glucose conjugate; **B** ^1^H NMR spectral of the conjugate and its intermediate compound; **C** ^13^C NMR spectral of the conjugate and its intermediate compound; **D** HMBC spectrum of the conjugate; **E** Key HMBC correlations of the conjugate; **F** FT-IR spectrum of the conjugate

The synthesis of the cholesterol-undecanoate-glucose conjugate is outlined in Fig. 5A. Glucose is a polyhydroxy compound, and there are multiple hydroxyl groups involved in its synthesis, which increases the difficulty of its purification. Therefore, an arbutin-containing fragment of glucose was reacted with bromine to generate cholesterol-undecanoate-glucose conjugate.

Cholesterol-undecanoate-glucose conjugate was a white amorphous powder, soluble in ethanol and tetrahydrofuran. ESI–MS showed *m/z*: 847.6 [M + Na]^+^ (Additional file 1: Fig. S13), suggesting its molecular formula is C_50_H_80_O_9_; infrared spectrum showed that the compound had a hydroxyl characterization absorption of 3,395 cm^−1^, a saturated alkane characteristic absorption of 2932 cm^−1^ and the characteristic absorption of ester carbonyl at 1735 cm^−1^ (Fig. 5F). ^1^H NMR spectrum showed *δ*_H_ 0.69 (3H, s, H-18), 1.03 (3H, s, H-19), 0.99 (3H, d, *J* = 6.4 Hz, H-21), 0.91 (6H, d, *J* = 6.6 Hz, H-26, 27), 4.88 (1H, m, H-3) were the characteristic proton signal of the four corner methyl groups of cholesterol and H-3. The ^13^C NMR data of cholesterol structural fragments were as follows: *δ*_C_ 37.8 (C-1), 29.0 (C-2), 74.4 (C-3), 39.1 (C-4), 140.5 (C-5), 123.3 (C-6), 32.5 (C-7), 32.7 (C-8), 50.8 (C-9), 37.3 (C-10), 21.8 (C-11), 40.2 (C-12), 43.0 (C-13), 57.3 (C-14), 25.0 (C-15), 28.7 (C-16), 56.9 (C-17), 12.5 (C-18), 19.9(C-19), 36.5 (C-20), 19.5 (C-21), 37.0 (C-22), 24.7 (C-23), 40.4 (C-24), 28.7 (C-25), 23.5 (C-26), 23.2 (C-27), the above-mentioned spectral data suggested that the cholesterol-undecanoate-glucose conjugate molecule had cholesterol structural fragments. In the ^1^H NMR spectrum, *δ*_H_ 2.43 (2H, t, *J* = 7.4 Hz, H-2'), 1.72 (2H, m, H-3'), 1.74 (2H, m, H-4'), 1.25–1.28 (8H, m, H-5'-8'), 1.43 (2H, m, H-9'), 1.75 (2H, m, H-10'), 3.89 (2H, t, *J* = 6.5 Hz, H -11') were the characteristic signal of undecanoate proton. The corresponding ^13^C NMR data were as follows: *δ*_C_ 173.5 (C-1'), 35.3 (C-2'), 25.9 (C-3'), 29.9 (C-4'), 28.7 (C-5'), 30.0 (C-6'), 30.2 (C-7'), 30.1 (C-8'), 26.8 (C-9'), 30.2 (C-10'), 69.1 (C-11'); The above spectral data suggested that the cholesterol-undecanoate-glucose conjugate molecule had undecanoate structural fragments. ^1^H NMR spectrum showed *δ*_H_ 7.38 (2H, d, *J* = 9.0 Hz, H-2'', H-6'') and 6.99 (2H, d, *J* = 9.0 Hz, H-3'', H-5' ') were the para-substituted aromatic ring proton signal, *δ*_H_ 5.53 (1H, d, *J* = 7.5 Hz, H-G-1), 4.29 (1H, m, H-G-2), 4.08 (1H, m, H-G-3), 4.33 (1H, m, H-G-4), 4.34 (1H, m, H-G-5), 4.54 (1H, d, *J* = 11.6 Hz, H-G-6a), 4.39 (1H, d, *J* = 11.6 Hz, H-G-6b) were a set of proton signals of glucose fragments. The corresponding ^13^C NMR data were as follows: *δ*_C_ 153.2 (C-1''), 118.8 (C-2'', 6''), 116.2 (C-3'', 5''), 155.3 (C- 4''), 103.8 (G-1), 75.5 (G-2), 79.3 (G-3), 71.8 (G-4), 79.0 (G-5), 62.9 (G-6). The above spectral data suggested that the molecule of cholesterol-undecanoate-glucose conjugate had arbutin structural fragment.

Through the analysis of the above-mentioned spectral data, it could be determined that the structural fragment of the cholesterol-undecanoate-glucose conjugate had a series of structural features of the target product. In order to clarify the connection mode of each structural fragment and the attribution of the hydrocarbon signal, two-dimensional nuclear magnetic spectroscopy HSQC, HMBC and ^1^H-^1^H COSY tests were further carried out. In the HMBC spectrum, the *δ*_H_ 4.88 (1H, m, H-3) of the cholesterol structure fragment was remotely related to the *δ*_C_ 173.5 (C-1') of the undecanoate structure fragment, indicating that the eleventh alkanoate structural fragments were connected in the form of ester bonds through C-1' and C-3 of cholesterol. In addition, the HMBC spectrum showed that the *δ*_H_ 3.89 (2H, t, *J* = 6.5 Hz, H-11') in the undecanoate structure fragment and the *δ*_C_ 155.3 (C -4''). There was a long-distance correlation, indicating that the undecanoate structural fragment was connected in the form of an ether bond through C-1' and the C-4" of the arbutin structural fragment (Fig. 5B–E). Combined with the analysis of the HMBC spectrum, it can be determined that the cholesterol-undecanoate-glucose conjugate was the target product, namely: cholesterol-undecanoate-glucose conjugate.

### Characterizations of liposomes

**Fig. 6:**
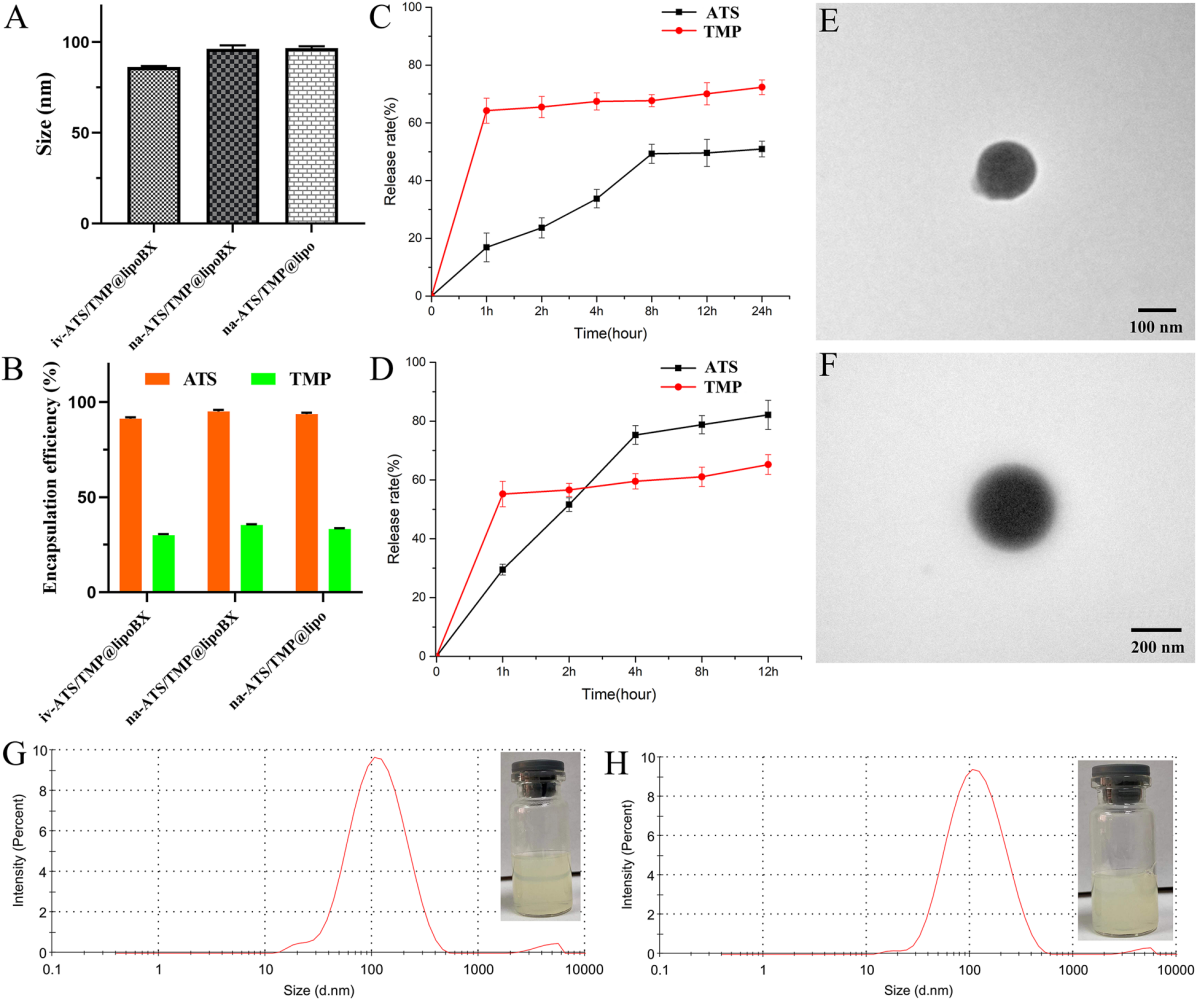
Characterizations of liposomes. **A** The average size of liposomes; **B **Encapsulation efficiency of liposomes; **C **and **D** Drugs cumulative release curves of na-ATS/TMP@lipoBX and iv-ATS/TMP@lipoBX, respectively. **E**, **F** TEM images of na-ATS/TMP@lipo and na-ATS/TMP@lipoBX, respectively. **G**, **H** Particle size distribution of na-ATS/TMP@lipo and na-ATS/TMP@lipoBX, respectively

The sizes of na-ATS/TMP@lipoBX, iv-ATS/TMP@lipoBX and na-ATS/TMP@lipo were 86.2 ± 0.9 nm, 96.2 ± 2.0 nm and 96.7 ± 1.1 nm, respectively (Fig. 3A). Through the observation of TEM, the liposomes showed a smooth spherical shape (Fig. 6E, H and Additional file 1: Fig. S15). The appearance of liposomes was translucent (Fig. 6G, H and Additional file 1: Fig. S16). The DSC spectrum of iv-ATS/TMP@lipoBX was shown in Additional file 1: Fig. S17.

Compared to na-ATS/TMP@lipoBX and na-ATS/TMP@lipo, the liposomes (na-ATS/TMP@lipoBX) modified by the conjugate have no significant changes in their characterizations. The result could indirectly verify the cholesterol-undecanoate-glucose conjugate can form a stable bilayer structure with phospholipid, which is a conclusion of molecular dynamics simulation.

### The encapsulation efficiency of liposomes

The encapsulation efficiency of ATS and TMP in the na-ATS/TMP@lipoBX and iv-ATS/TMP@lipoBX were measured by high-performance liquid chromatography (HPLC). The encapsulation efficiency of ATS in the three kinds of liposomes was higher than 90%, and the encapsulation efficiency of TMP was close to 30% (Fig. 6B).

### In vitro drug release

The in vitro release rate is an essential parameter for control of drug quality. The cumulative release rates of ATS from iv-ATS/TMP@lipoBX and na-ATS/TMP@lipoBX were about 80% and 50% in the first 8 h, respectively. The cumulative release rates of TMP from iv-ATS/TMP@lipoBX and na-ATS/TMP@lipoBX were about 65% and 55% in the first 1 h, respectively (Fig. 6C, D). The release of ATS and TMP from the above two liposomes could be classified into two phases: a rapid release phase during which unencapsulated ATS and TMP coordinated at the surface of the lipid bilayer might be released all at once. This initial phase was followed by a steady release, known as the slow release phase [58].

ATS was released from iv-ATS/TMP@lipoBX and na-ATS/TMP@lipoBX at different rates during the rapid release phase, and this result might be explained by the higher the phospholipid content in the na-ATS/TMP@lipoBX, which encapsulated ATS tighter. This release characterization of na-ATS/TMP@lipoBXg is beneficial to inhibit the recurrence of malaria.

### Stability study of liposomes

#### Placement stability of liposomes

**Fig. 7:**
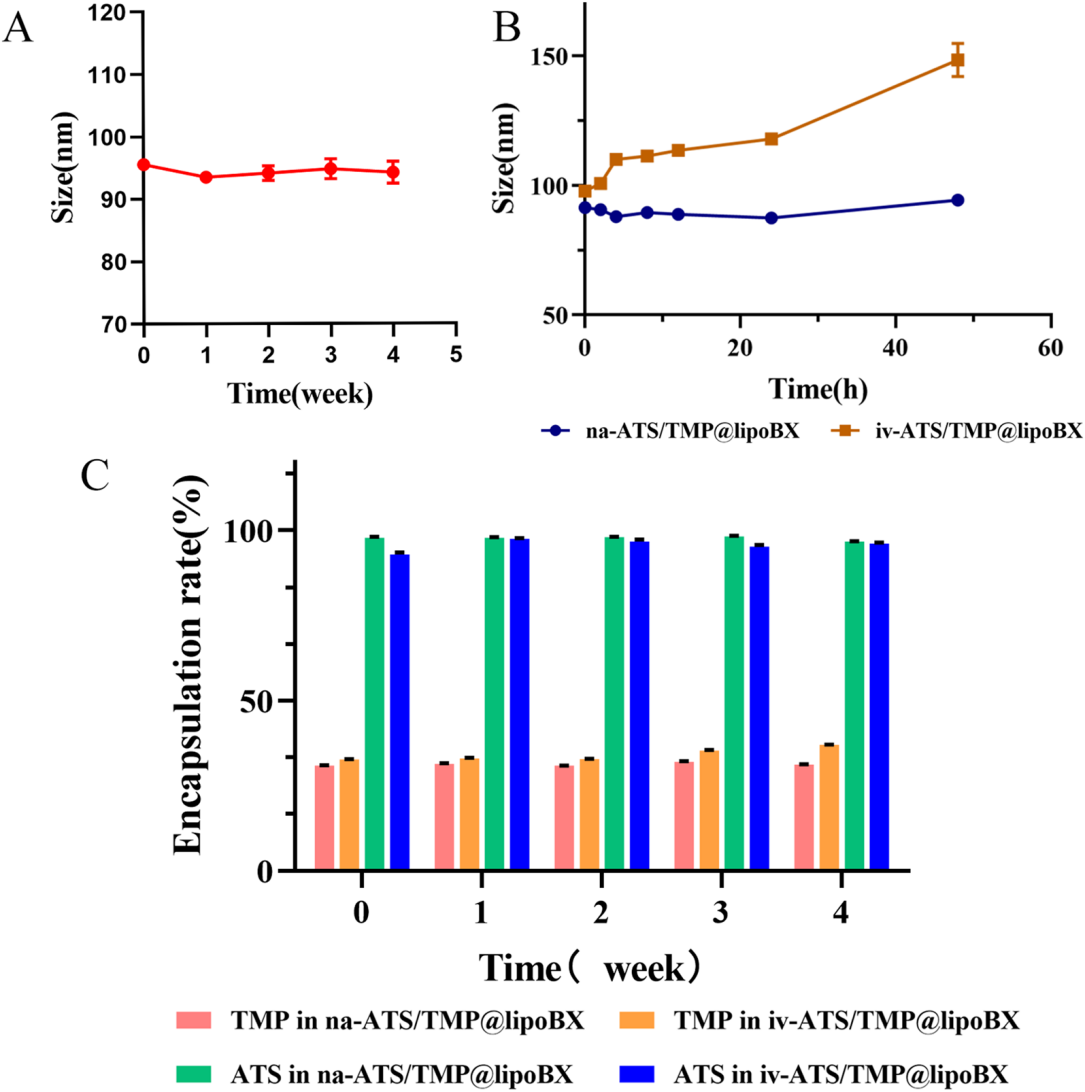
The liposome stability studies; **A** The size of liposomes in long-term storage stability; **B** The size of liposomes in serum; **C** The encapsulation efficiency of TMP and ATS in na-ATS/TMP@lipoBX and iv-ATS/TMP@lipoBX in long-term storage stability, respectively

The RSDs of particle size and encapsulation efficiency of na-ATS/TMP@lipo at weeks 0,1,2,3, and 4 were as follows: 0.98% and 0.59% (ATS), 1.50% (TMP). The RSDs of particle size and encapsulation efficiency of iv-ATS/TMP@lipoBX at weeks 0,1,2,3 and 4 were as follows: 0.82% and 1.82% (ATS), 5.61% (TMP) (Fig. 7A–C).

#### Stability of liposomes in serum

The particle size RSDs of na-ATS/TMP@lipo within 24 h and 48 h in serum were as follows:1.72% and 2.63%; the particle size RSDs of iv-ATS/TMP@lipoBX within 24 h and 48 h in serum were as follows:7.11% and 15.54% (Fig. 7B).

### In vivo brain-targetability evaluation

#### Nasal administration

**Fig. 8:**
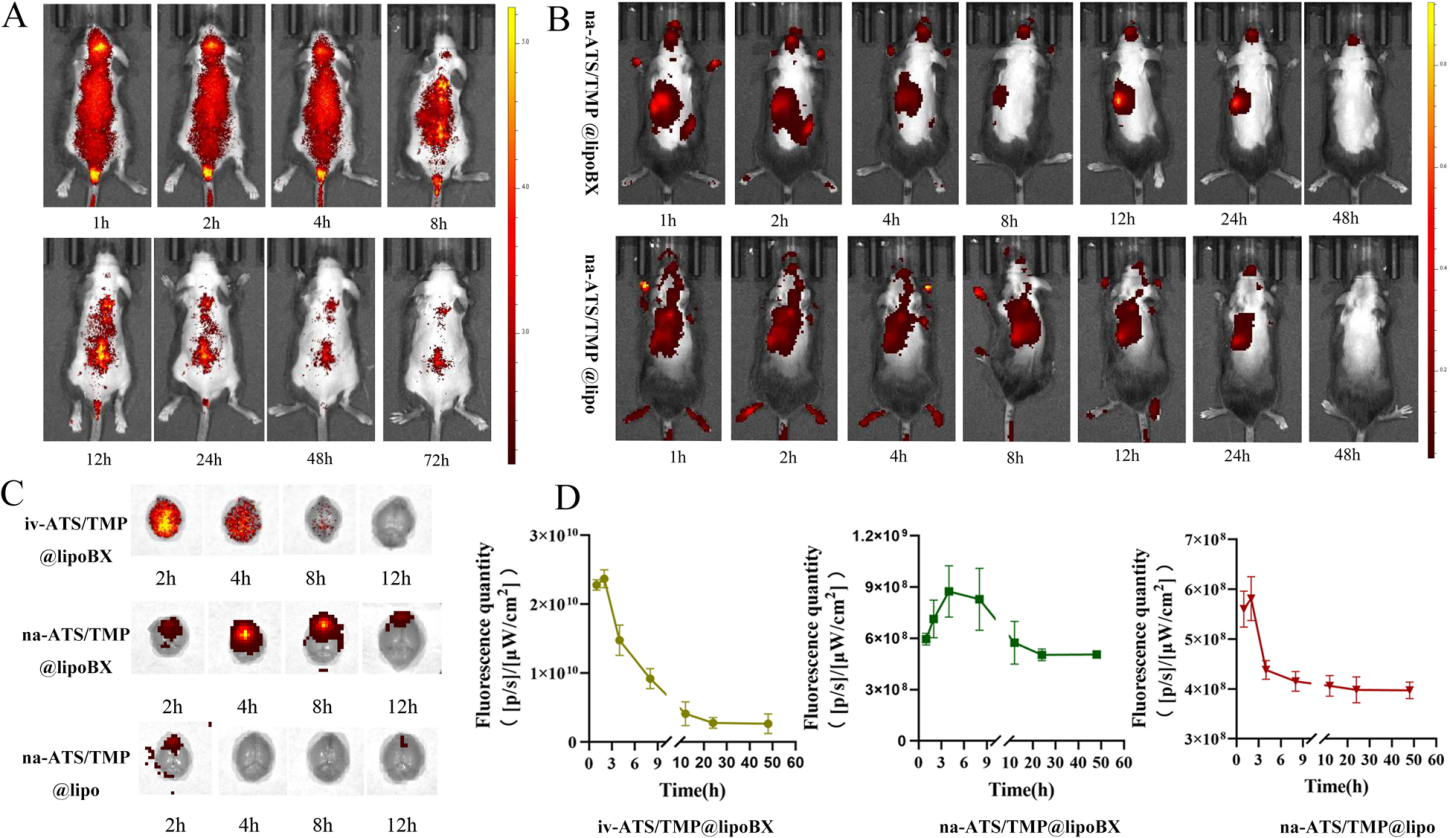
**A** The fluorescence images of mice treated with iv-DiR@ATS/TMP@lipoBX (brain-targeted liposomes). **B** The fluorescence images of mice treated with na-DiR@ATS/TMP@lipoBX and na-DiR@ATS/TMP@lipo (non-targeted liposomes), respectively. **C** The fluorescence images of the brain of mice treated with iv-DiR@ATS/TMP@lipoBX, na-DiR@ATS/TMP@lipoBX and na-DiR@ATS/TMP@lipo, respectively; **D** the Fluorescence quantity of iv-DiR@ATS/TMP@lipoBX, na-DiR@ATS/TMP@lipoBX and na-DiR@ATS/TMP@lipo, respectively (n = 3)

The distribution of the liposomes in the mice was evaluated dynamically using a DiR fluorescent probe. The results showed that na-DiR@ATS/TMP@lipoBX (brain-targeted liposomes) had a longer retention time in the brain compared to na-DiR@ATS/TMP@lipo (non-targeted liposomes) (Fig. 8B). At 48 h after administration, a certain intensity of fluorescence signal could still be detected in the isolated brain of mice in the targeted administration group, while the fluorescence intensity in the brain of the non-targeted administration group decreased rapidly at 4 h after administration (Fig. 8D).

The effects of in vivo brain fluorescence imaging and ex vivo brain fluorescence imaging was compared in the study. In the in vivo fluorescence imaging results, obvious fluorescence signals could be seen in the mouse head 1 h after administration, but the brain ex vivo fluorescence imaging results showed that the targeted liposomes entered the brain with a certain delay. The fluorescence intensity in the brain reached the maximum value 4 h after administration (Fig. 8C).

In brain, fluorescence imaging showed that liposomes first reached the olfactory bulb after nasal administration. Some studies have shown that cerebral malaria can cause hemorrhage and inflammation in the olfactory bulb [59–61], so brain-targeted liposomes can deliver drugs to the olfactory bulb to achieve targeted therapy. The efficacy evaluation results in this paper verified the advantages of na-DiR@ATS/TMP@lipoBX in the treatment of cerebral malaria.

#### Injection administration

After iv-ATS/TMP@lipoBX injection, the fluorescence signal could be detected rapidly in the isolated brain, but the fluorescence intensity decreased significantly over time, and there was no obvious fluorescence signal in the isolated brain after 8 h.

Compared with intranasal administration of DiR@ATS/TMP@lipoBX, the injection of iv-ATS/TMP@lipoBX had a shorter retention time in the brain, which affected the antimalarial efficacy (Fig. 8A).

### Modeling cerebral malaria

**Fig. 9:**
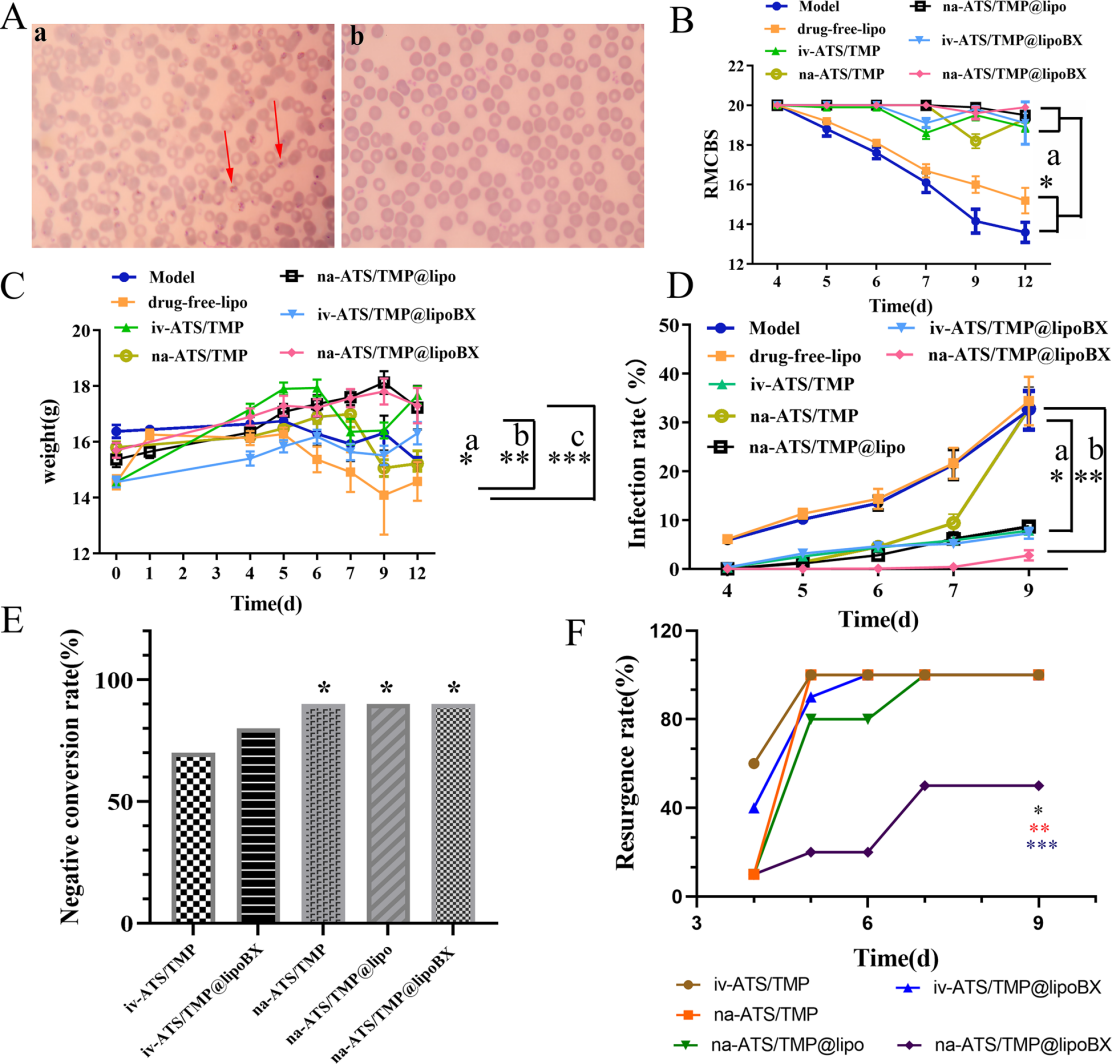
**A** The red cells of healthy mice and mice infected with plasmodium (100 × oil microscope), **a**: red cells of mice infected with plasmodium, **b**: normal red cells; **B**–**F** Charts are RMCBS, weight, infected rate, negative conversion and recurrence rate of mice after treated with drugs, respectively. *P < 0.05, **P < 0.01, ***P < 0.001. In **E**, * represents na-ATS/TMP, na-ATS/TMP@lipo, na-ATS/TMP@lipoBX compare with iv-ATS/TMP. In **F**, * represents na-ATS/TMP@lipoBX compare with na-ATS/TMP, na-ATS/TMP@lipo. ** represents na-ATS/TMP@lipoBX compare with iv-ATS/TMP@lipoBX. *** represents na-ATS/TMP@lipoBX compare with iv-ATS/TMP

Smears of red blood cells obtained from healthy mice and mice infected with *P. falciparum* are shown in Fig. 9A. The red blood cells in the healthy mice were uniform in size, shaped like a double concave disc, and harbored no abnormal structures in the cytoplasm. The red blood cells infected with *P. falciparum* had the shape of rings, trophozoites and schizonts (red arrow), which indicated that the modeling was successful.

### Effect of drugs on rapid murine coma and behavior scale (RMCBS) of mice

When observing the behavior of mice during exercise and exploration, their anxiety, depression, and other symptoms can reflect the effect of drugs. Mice in the model and drug-free lipo group began to show unsteady walking, decreased limb strength, and a loss of fur gloss by the 5th day of the experiment, while their RMCBS showed a downward trend throughout the experiment. The RMCBS of the other groups showed a slight downward trend during the observation period. Compared with the model and drug-free lipo groups, there was a significant difference (P < 0.05). The na-ATS/TMP group showed a significant decrease in RMCBS on the 9th day, possibly because the small nasal area of the mice absorbed an insufficient amount of the drug, and the solution lacked a liposome-like sustained-release effect, which allowed the drug to clear from the body rapidly. On the 12th day, the RMCBS of the na-ATS/TMP@lipoBX group was higher than that in the other administration groups (Fig. 9B).

### Effect of drugs on weight of mice

The body weights of mice in the model, drug-free lipo and na-ATS/TMP (intranasal administration of ATS and TMP solutions) groups during the observation period first increased and then decreased. The body weights of the mice in the na-ATS/TMP@lipo groups showed an increasing trend that reached a maximum on the 9th day; during the observation period, the body weights of mice in the iv-ATS/TMP (intravenous administration of ATS and TMP solutions) and iv-ATS/TMP@lipoBX groups showed a trend of first increasing, then decreasing and then increasing. The body weights of mice in the na-ATS/TMP@lipo and the iv-ATS/TMP groups were higher than those of the model group (P < 0.05) and significantly higher than those in the drug-free lipo group (P < 0.01) (Fig. 9C).

### Effect of drugs on infection rate of mice

The infection rate is an important indicator for evaluating drug efficacy, and in this case, the infection rate is determined by the number of red blood cells infected by *P. falciparum* divided by the total number of red blood cells (either 1000 or more than 1000 blood red cells). In this study, the therapeutic effects of the solution groups and the liposome groups on the cerebral malaria model mice after injection and intranasal administration were evaluated. Table 1 shows that the infection rate of the na-ATS/TMP@lipoBX (brain-targeted liposomes) group was significantly lower than that of the other administration groups, and the infection rate on the 9th day of observation was only 3.34%. This was significantly lower than the model, drug-free lipo and na-ATS/TMP groups (P < 0.01) and lower than the na-ATS/TMP@lipo, iv-ATS/TMP@lipoBX and iv-ATS/TMP groups (P < 0.05). There was no significant difference between the infection rate of the model and drug-free lipo groups during the observation period (P > 0.05), indicating that drug-free lipo had no pharmacological effects (Fig. 9D). These results showed that brain-targeted based on GLUT1 could significantly enhance the therapeutic effect against cerebral malaria.

**Table 1:** Effects of drugs on the infection rate (%) of mice

Group	Infection rate (%)				
	4th day	5th day	6th day	7th day	9th day
Model	5.74	9.69	12.80	20.07	29.92
drug-free lipo	5.92	10.74	13.59	20.33	32.09
iv-ATS/TMP	0.60	2.80	4.53	5.96	7.96
iv-ATS/TMP@lipoBX	0.66	3.30	4.76	5.33	7.47
na-ATS/TMP@lipoBX	0.56	0.47	0.65	1.01	3.34
na-ATS/TMP	0.01	1.34	4.50	9.40	32.8
na-ATS/TMP@lipo	0.01	1.14	2.80	6.10	8.70

### Effects of drugs on negative conversion rate of mice

The negative conversion rate was indicated by no *P. falciparum* or an infection rate < 0.5% for each mouse after stopping administration for 24 h, and the negative mice were classified by the total mice in each group. The negative conversion rate of the iv-ATS/TMP group was 70%, that of the na-ATS/TMP group was 90%. And the negative conversion rate of the iv-ATS/TMP@lipoBX group was 80%, the negative conversion rates of na-ATS/TMP@lipoBX and na-ATS/TMP@lipo groups were both 90% (Fig. 9E). According to the above, the negative conversion rates of the intranasal administration groups were better than that of the injection groups.

### Effect of drugs on recurrence rate of malaria

The recurrence rate of the negative mice after stopping administration was evaluated. The na-ATS/TMP@lipoBX (brain-targeted liposomes) showed significant advantages in preventing the recurrence of cerebral malaria. Cerebral malaria recurred in all mice of the injection (iv-ATS/TMP@lipoBX and iv-ATS/TMP) groups on the 6th day after stopping administration. Cerebral malaria recurred in all mice of the na-ATS/TMP@lipo groups on the 7th day after stopping administration. However, the mice in the na-ATS/TMP@lipoBX group had a lower recurrence rate, only 50% of mice have a resurgence of malaria on the 9th day after stopping administration (Fig. 9F).

The results of this study are consistent with the results of in *vivo* brain-targetability evaluation. After nasal administration, brain-targeted liposomes (na-ATS/TMP@lipoBX) could be effectively enriched in the brain and prolong the retention time of the drug in the brain with the help of cholesterol-undecanoate-glucose conjugate. As a result, brain-targeted liposomes could effectively decrease the resurgence rate of cerebral malaria. It is of great significance to the treatment of cerebral malaria.

### Pharmacokinetic and brain biodistribution studies

**Fig. 10:**
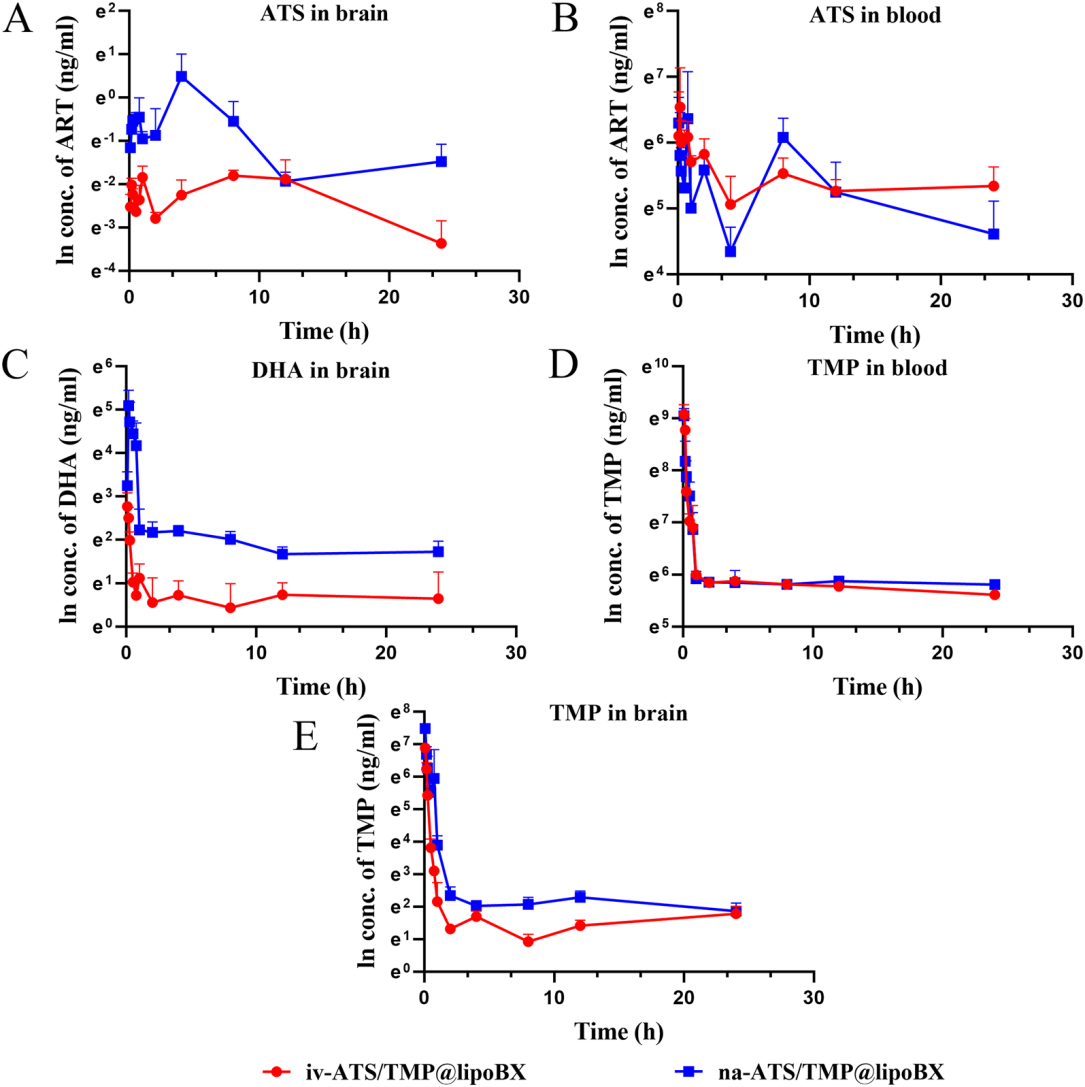
The concentration curve of ATS and TMP in plasma and brain versus time after i.n. administration of na-ATS/TMP@lipoBX or i.v. administration of iv-ATS/TMP@lipoBX in mice. The concentration curve of DHA in brain versus time after i.n. administration of na-ATS/TMP@lipoBX or i.v. administration of iv-ATS/TMP@lipoBX in mice. Data represent the mean ± SD (n = 4)

The concentration curves and pharmacokinetic parameters of ATS, TMP and dihydroartemisinin (artesunate metabolites [62]) in plasma or brain after intranasal (i.n.) administration of na-ATS/TMP@lipoBX and intravenous (i.v.) administration of na-ATS/TMP@lipoBX in mice are shown in Fig. 10 and Table 2. The results showed that na-ATS/TMP@lipoBX after i.n. administration could enter the systemic circulation in large quantities. The relative bioavailabilities of ATS and TMP in plasma by i.n. administration was approximately was 91% and 97%, respectively, compared with that of i.v. administration. This indicates that the targeted liposomes could deliver drugs to the brain across the blood–brain barrier via this pathway.

**Table 2:** Pharmacokinetic and brain biodistribution parameters of ATS, TMP and DHA (n = 4)

Compounds	Administration	Samples	AUC_(0-t)_ (μg/L·h)	C_max_ (μg/L)	T_max_ (h)	MRT_(0-t)_ (h)
ATS	i.n	Plasma	4915.556 ± 1413.94	879.559 ± 309.656	2.396 ± 3.749	9.801 ± 1.556
		Brain	11.128 ± 3.797	1.663 ± 1.070	3.500 ± 1.000	7.895 ± 0.299
	i.v	Plasma	5387.243 ± 529.557	938.105 ± 444.814	0.292 ± 0.308	11.039 ± 1.269
		Brain	2.684 ± 0.711	0.208 ± 0.023	8.250 ± 5.188	9.764 ± 0.736
TMP	i.n	Plasma	10,144.79 ± 461.39	8521.69 ± 1283.845	0.083 ± 0.000	9.79 ± 0.393
		Brain	682.381 ± 95.191	1794.855 ± 213.922	0.083 ± 0.000	3.583 ± 0.555
	i.v	Plasma	10,431.609 ± 312.259	8917.914 ± 1704.089	0.104 ± 0.042	8.376 ± 0.249
		Brain	372.172 ± 20.836	980.815 ± 130.674	0.083 ± 0.000	3.884 ± 0.404
DHA	i.n	Brain	224.205 ± 19.141	175.042 ± 54.372	0.396 ± 0.283	7.683 ± 0.829
	i.v	Brain	49.942 ± 14.06	16.797 ± 5.249	0.125 ± 0.048	10.915 ± 2.593

*ATS*: artesunate, *TMP* tetramethylpyrazine hydrochloride, *DHA* dihydroartemisinin, *i.n.* intranasal, *i.v.* intravenous

After i.n. administration of na-ATS/TMP@lipoBX, the concentration of the drug in the brain was significantly higher than that in the i.v. administration group. The relative bioavailabilities of ATS and TMP in the brain by i.n. administration was approximately 415% and 183%, respectively, compared with that of i.v. administration. Based on this result, it could be speculated that targeted liposomes could also deliver drugs directly to the brain through the nasal-brain pathway [63]. The in vivo imaging results (In vivo brain-targetability evaluation) of na-ATS/TMP@lipoBX verified this hypothesis, and the study showed that na-ATS/TMP@lipoBX could aggregate at the olfactory bulb to achieve direct brain-targeted delivery.

In addition, ATS could be metabolized in the body into another antimalarial active drug, dihydroartemisinin (DHA) [62]. The concentration of DHA in the brain was quantitatively analyzed, the concentration curve and pharmacokinetic parameters of DHA in brain after i.n. administration of na-ATS/TMP@lipoBX or i.v. administration of iv-ATS/TMP@lipoBX was shown in Fig. 10C and Table 2. After i.n. administration, the C_max_ and AUC _(0-t)_ of DHA were 10.4 and 4.5 times those of i.v. administration, respectively. The relative bioavailability of DHA in brain by i.n. administration was approximately 449% compared with that of i.v. administration. A higher concentration of DHA delivered to the brain could kill the malaria parasite in brain more effectively.

The above results showed that na-ATS/TMP@lipoBX could enter the brain in two ways (systemic circulation and nasal-brain pathways) to produce antimalarial effects after i.n. administration and the liposomes had an ideal brain-targeted drug delivery ability by i.n. administration.

### Histological evaluation.

**Fig. 11:**
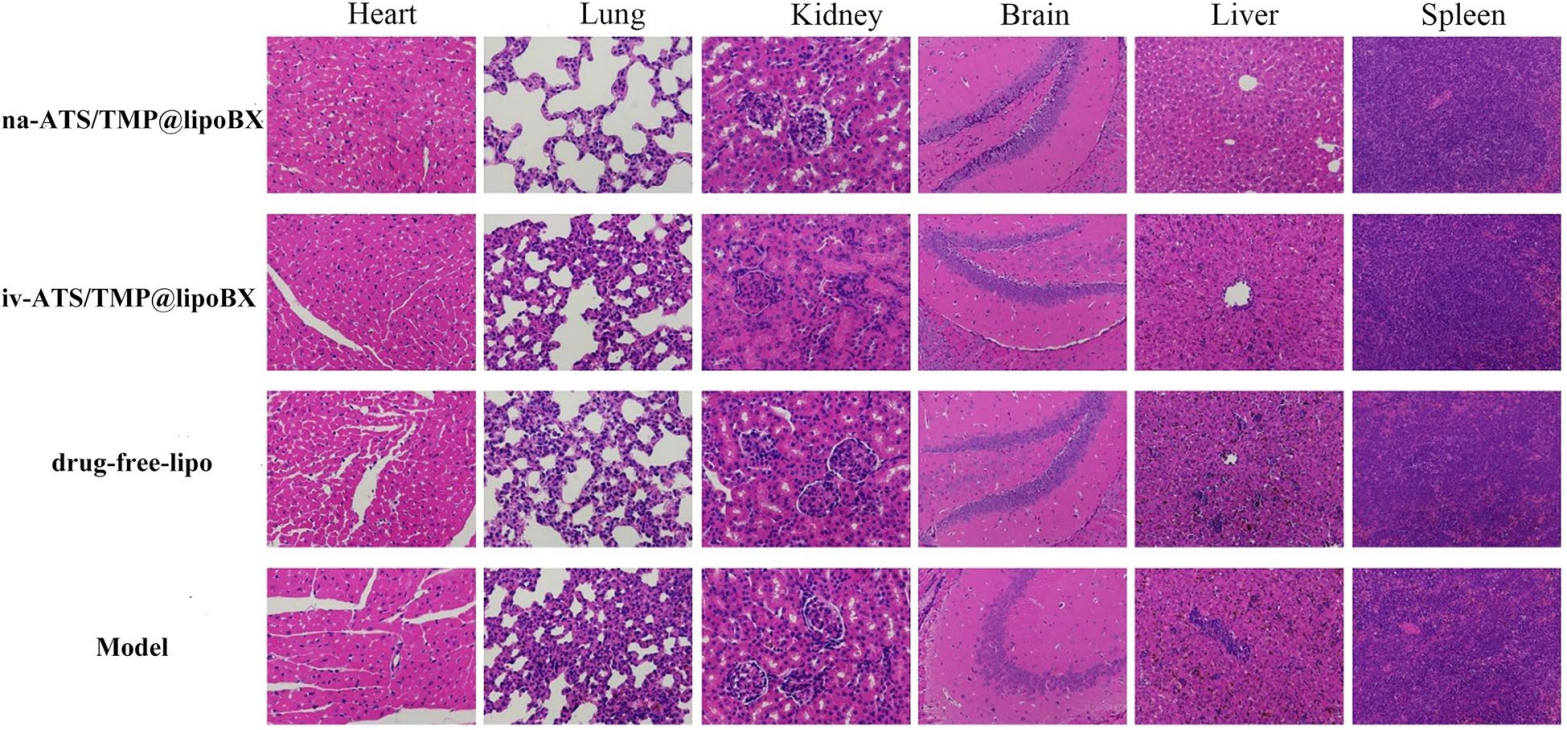
The H&E staining of heart, lungs kidney, brain, liver and spleen, respectively (Heart, lung and kidney are 400 × magnification; brain, liver and spleen are 200 × magnification)

The pathological results of each administration group are shown in Fig. 11. No obvious histological abnormalities were caused by the nano-preparations in any of the main organs of the mice after intranasal administration of na-ATS/TMP@lipoBX (brain-targeted liposomes). This result shows that na-ATS/TMP@lipoBX has good biosafety.

In addition, compared with the intranasal administration group, the alveolar walls of the mice in the tail vein injection groups (iv-ATS/TMP@lipoBX group and drug-free lipo group) and model group were significantly thicker; the model and drug-free lipo groups were thought to show pathological changes in response to *P. falciparum* invasion. In the intranasal administration of na-ATS/TMP@lipoBX group, the nuclei of liver cells stained more evenly, and the liver cells were arranged radially around the central vein (Fig. 11). The above-mentioned pathological results also showed that na-ATS/TMP@lipoBX had certain advantages in the treatment of cerebral malaria in mice.

## Conclusions

Through molecular dynamics simulation of phospholipid bilayer formation, it was found that the cholesterol-undecanoate-glucose conjugate was stably inserted into the phospholipid bilayer, and the structural fragment of glucose in the conjugate faced outside the phospholipid bilayer, which was conducive to the recognition of brain-targeted liposomes by GLUT1. In-depth molecular dynamics simulation studies showed that the conjugate did not affect important parameters of the bilayer structure, which were area per lipid, deuteration order of the hydrocarbon chains and electron density. This result provided a theoretical basis for the follow-up study of the brain-targeted nano-drug delivery system. In a further study, the cholesterol-undecanoate-glucose conjugate as the GLUT1 targeted material was synthesized by a two-step condensation reaction. The liposomes prepared by the thin-film dispersion method had good morphology and ideal uniformity of particle size. The encapsulation efficiency of ATS and TMP were approximately 90% and 30%, respectively. The in vitro drug release experiments showed that the liposome for drug delivery could control drug release and no burst release phenomenon was observed. Compared with non-targeted liposomes without glucose modification, brain-targeted liposomes (na-ATS/TMP@lipoBX) significantly enhanced brain fluorescence intensity in mice. Furthermore, the infection and recurrence rate of the mice receiving na-ATS/TMP@lipoBX treatment was significantly decreased, which had more advantages than those of other administration groups. The analysis of pharmacokinetic data in vivo showed that na-ATS/TMP@lipoBX (brain-targeted liposomes) could enter the brain in two ways (systemic circulation and nasal-brain pathways) to produce antimalarial effects after i.n. administration and the liposomes had an ideal brain-targeted drug delivery ability by i.n. administration. Finally, pathological study showed na-ATS/TMP@lipoBX had good biosafety. The study suggests a new approach to the treatment of cerebral malaria.

## Supplementary Information

**Additional file 1: Figure S1** The chemical structure of 3-(11-bromoundecanoate)-cholesterol. **Figure S2** Snapshots showing the spontaneous self-assembly of PLPC into a bilayer. The PLPC and undecane-glucose conjugate is depicted as yellow and cyan respectively, blue is nitrogen atom in phospholipids, red is oxygen atom in conjugates. Note that water molecules are not shown. **Figure S3** Snapshots showing the spontaneous self-assembly of PLPC into a bilayer. The PLPC and arbutin is depicted as yellow and cyan respectively, blue is nitrogen atom in phospholipids, red is oxygen atom in conjugates. Note that water molecules are not shown. **Figure S4** Snapshots showing the spontaneous self-assembly of PLPC into a bilayer. The PLPC and cholesterol is depicted as yellow and cyan respectively, blue is nitrogen atom in phospholipids, red is oxygen atom in conjugates. Note that water molecules are not shown. **Figure S5** ^1^H NMR spectrum of 3-(11-bromoundecanoate)-cholesterol. **Figure S6** ^13^C NMR spectrum of 3-(11-bromoundecanoate)-cholesterol. **Figure S7** Infrared spectrum of 3-(11-bromoundecanoate)-cholesterol. **Figure S8** ^1^H NMR spectrum of cholesterol-undecanoate-glucose conjugate. **Figure S9** ^13^C NMR spectrum of cholesterol-undecanoate-glucose conjugate. **Figure S10** COSY spectrum of Cholesterol-undecanoate-glucose conjugate. **Figure S11** HSQC spectrum of Cholesterol-undecanoate-glucose conjugate. **Figure S12** HMBC spectrum of Cholesterol-undecanoate-glucose conjugate. **Figure S13** ESI mass spectrum of Cholesterol-undecanoate-glucose conjugate. **Figure S14** Infrared spectrum of Cholesterol-undecanoate-glucose conjugate. F**igure S15** is TEM image of iv-ART/TMP@lipoBX **Figure S16** Particle size distribution of iv-ART/TMP@lipoBX. **Figure S17** DSC spectrum of iv-ART/TMP@lipoBX.

## Data Availability

Not applicable.
