# Supplementary material for: Establishment and evaluation of glucose-modified nanocomposite liposomes for the treatment of cerebral malaria
Source: J Nanobiotechnology. 2022 Jul 6;20:318. doi: 10.1186/s12951-022-01493-8 (PMC9258070; doi:10.1186/s12951-022-01493-8)
Supplement: Supplementary file 1 — Additional file 1: Figure S1 The chemical structure of 3-(11-bromoundecanoate)-cholesterol. Figure S2 Snapshots showing the spontaneous self-assembly of PLPC into a bilayer. The PLPC and undecane-glucose conjugate is depicted as yellow and cyan respectively, blue is nitrogen atom in phospholipids, red is oxygen atom in conjugates. Note that water molecules are not shown. Figure S3 Snapshots showing the spontaneous self-assembly of PLPC into a bilayer. The PLPC and arbutin is depicted as yellow and cyan respectively, blue is nitrogen atom in phospholipids, red is oxygen atom in conjugates. Note that water molecules are not shown. Figure S4 Snapshots showing the spontaneous self-assembly of PLPC into a bilayer. The PLPC and cholesterol is depicted as yellow and cyan respectively, blue is nitrogen atom in phospholipids, red is oxygen atom in conjugates. Note that water molecules are not shown. Figure S5 1H NMR spectrum of 3-(11-bromoundecanoate)-cholesterol. Figure S6 13C NMR spectrum of 3-(11-bromoundecanoate)-cholesterol. Figure S7 Infrared spectrum of 3-(11-bromoundecanoate)-cholesterol. Figure S8 1H NMR spectrum of cholesterol-undecanoate-glucose conjugate. Figure S9 13C NMR spectrum of cholesterol-undecanoate-glucose conjugate. Figure S10 COSY spectrum of Cholesterol-undecanoate-glucose conjugate. Figure S11 HSQC spectrum of Cholesterol-undecanoate-glucose conjugate. Figure S12 HMBC spectrum of Cholesterol-undecanoate-glucose conjugate. Figure S13 ESI mass spectrum of Cholesterol-undecanoate-glucose conjugate. Figure S14 Infrared spectrum of Cholesterol-undecanoate-glucose conjugate. Figure S15 is TEM image of iv-ART/TMP@lipoBX Figure S16 Particle size distribution of iv-ART/TMP@lipoBX. Figure S17 DSC spectrum of iv-ART/TMP@lipoBX. [file 12951_2022_1493_MOESM1_ESM.docx]

**Supporting Information for original article**

**Establishment and evaluation of glucose-modified nanocomposite liposomes for the treatment of cerebral malaria**

**Ya Tian^a,b†^, Zhongyuan Zheng^a†^, Xi Wang^a^, Shuzhi Liu^a^, Liwei Gu^a^, Jing Mu^c^, Xiaojun Zheng^d^, Yujie Li^a,^* and Shuo Shen^a,^***

^a^*Institute of Chinese Materia Medica, China Academy of Chinese Medical Sciences, Beijing* *100700, PR China*

*^b^The Hospital of Nanbu County, Sichuan, PR China*

*^c^Chinese Traditional Medicine Resource Center, China Academy of Chinese Medical Sciences, Beijing 100700, PR China.*

^d^*Pharmacy Department of the first hospital of Shanxi Medical University, Shanxi 10114, PR China*

Corresponding authors:

* Email for Shuo Shen: sshen@icmm.ac.cn

* Email for Yujie Li: yjli@icmm.ac.cn

1. ***Chemical structure analysis of 3-(11-bromoundecanoate)-cholesterol***

The structure of 3-(11-bromoundecanoate)-cholesterol is shown in **Figure S1**. It was a white amorphous powder and easily soluble in dichloromethane; infrared spectrum showed that the compound had a characteristic absorption of saturated alkanes of 2933 cm^-1^ and a characteristic absorption of ester carbonyl of 1736 cm^-1^ (**Figure S7**). ^1^H NMR spectrum showed *δ*_H_ 0.68 (3H, s, H-18), 1.02 (3H, s, H-19), 0.92 (3H, d, *J* = 6.5 Hz, H-21), 0.87 (3H, d, *J* = 6.7 Hz, H-26 or 27), 0.86 (3H, d, *J* = 6.6 Hz, H-26 or 27), 4.61 (1H, m, H-3) were the four corner methyl groups of cholesterol and H-3 characteristic proton signal. The ^13^C NMR data of cholesterol structural fragments were as follows: *δ*_C_ 37.2 (C-1), 29.5 (C-2), 73.9 (C-3), 39.9 (C-4), 139.9 (C-5), 122.8 (C-6 ), 32.1 (C-7), 32.1 (C-8), 50.2 (C-9), 36.8 (C-10), 21.2 (C-11), 39.7 (C-12), 42.5 (C-13) , 56.9 (C-14), 24.5 (C-15), 28.4 (C-16), 56.4 (C-17), 12.1 (C-18), 18.9 (C-19), 36.0 (C-20), 19.5 (C-21), 36.4 (C-22), 24.0 (C-23), 38.4 (C-24), 28.4 (C-25), 23.0 (C-26), 22.8 (C-27), above spectral data suggested that 3-(11-bromoundecanoate)-cholesterol molecule had cholesterol structural fragments. In the ^1^H NMR spectrum, *δ*_H_ 2.26 (2H, t, *J* = 7.5 Hz, H-2'), 3.40 (2H, t, *J* = 6.9 Hz, H-11') was the proton of 11 bromo-undecanoate characteristic signal. The ^13^C NMR data corresponding to this structural fragment were as follows: *δ*_C_ 173.4 (C-1'), 34.9 (C-2'), 25.2 (C-3'), 28.4 (C-4'), 29.4 (C-5'), 29.5 (C-6'), 28.9 (C-7'), 28.0 (C-8'), 28.2 (C-9'), 33.0 (C-10'), 34.1 (C-11'), the above-mentioned spectral data suggested that 3-(11-bromoundecanoate)-cholesterol molecule had 11 bromo-undecanoate structural fragments. Since the chemical shift value of the C-3 position of the cholesterol shifted to the low field by about 3 ppm, it could be inferred that 11 bromo-undecanoic acid and the 3-hydroxyl group of cholesterol were dehydrated and condensed to 3-(11-bromoundecanoate)-cholesterol (**Figure S1**).

1. ***Other charts are as follows***

**Figure S1**: The chemical structure of 3-(11-bromoundecanoate)-cholesterol

**Figure S2**: Snapshots showing the spontaneous self-assembly of PLPC into a bilayer. The PLPC and undecane-glucose conjugate is depicted as yellow and cyan respectively, blue is nitrogen atom in phospholipids, red is oxygen atom in conjugates. Note that water molecules are not shown.

**Figure S3**: Snapshots showing the spontaneous self-assembly of PLPC into a bilayer. The PLPC and arbutin is depicted as yellow and cyan respectively, blue is nitrogen atom in phospholipids, red is oxygen atom in conjugates. Note that water molecules are not shown.

**Figure S4**: Snapshots showing the spontaneous self-assembly of PLPC into a bilayer. The PLPC and cholesterol is depicted as yellow and cyan respectively, blue is nitrogen atom in phospholipids, red is oxygen atom in conjugates. Note that water molecules are not shown.

**Figure S5** ^1^H NMR spectrum of 3-(11-bromoundecanoate)-cholesterol.

**Figure S6** ^13^C NMR spectrum of 3-(11-bromoundecanoate)-cholesterol.

**Figure S7** Infrared spectrum of 3-(11-bromoundecanoate)-cholesterol.

**Figure S8** ^1^H NMR spectrum of cholesterol-undecanoate-glucose conjugate.

**Figure S9** ^13^C NMR spectrum of cholesterol-undecanoate-glucose conjugate.

**Figure S10** COSY spectrum of Cholesterol-undecanoate-glucose conjugate.

**Figure S11** HSQC spectrum of Cholesterol-undecanoate-glucose conjugate.

**Figure S12** HMBC spectrum of Cholesterol-undecanoate-glucose conjugate.

**Figure S13** ESI mass spectrum of Cholesterol-undecanoate-glucose conjugate.

**Figure S14** Infrared spectrum of Cholesterol-undecanoate-glucose conjugate.

**Figure S15** is TEM image of iv-ART/TMP@lipoBX.

**Figure S16** Particle size distribution of iv-ART/TMP@lipoBX.

**Figure S17** DSC spectrum of iv-ART/TMP@lipoBX.

**Figure S1**: The chemical structure of 3-(11-bromoundecanoate)-cholesterol


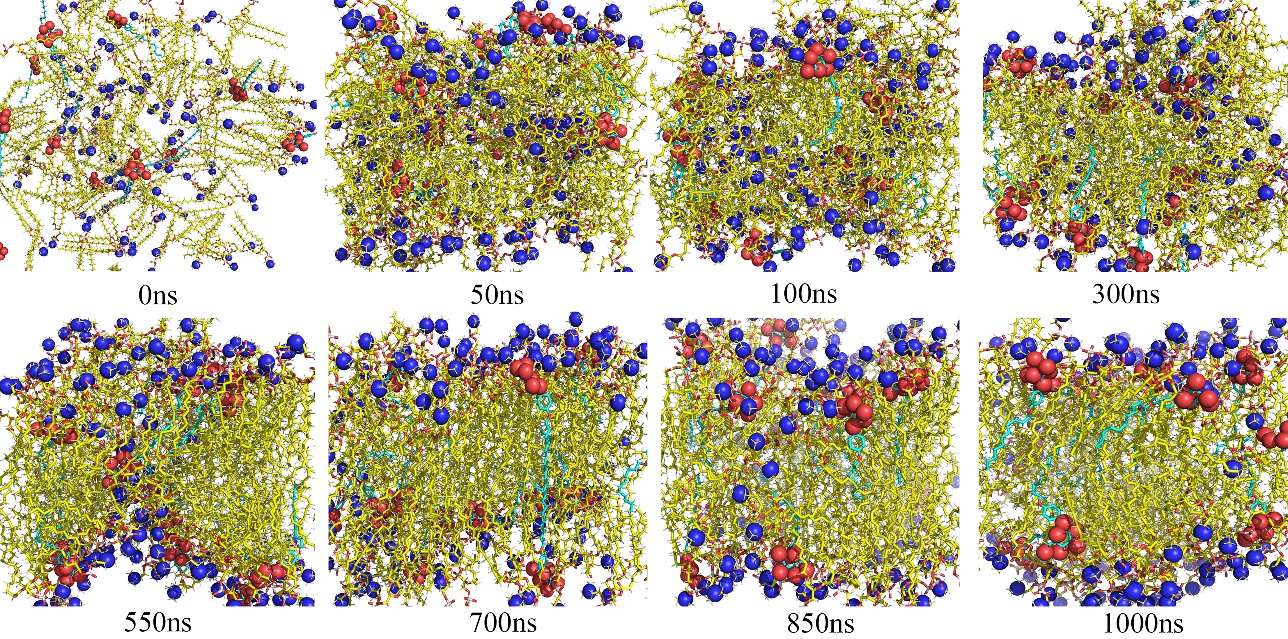


**Figure S2**: Snapshots showing the spontaneous self-assembly of PLPC into a bilayer. The PLPC and undecane-glucose conjugate is depicted as yellow and cyan respectively, blue is nitrogen atom in phospholipids, red is oxygen atom in conjugates. Note that water molecules are not represented.


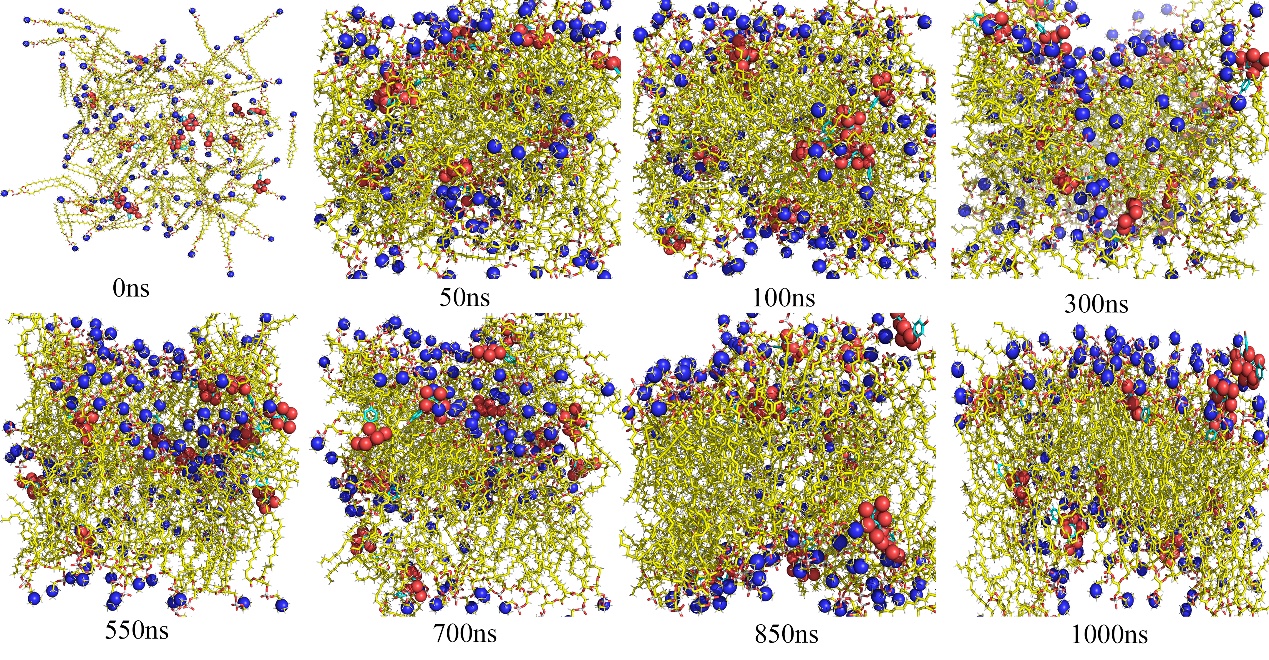


**Figure S3**: Snapshots showing the spontaneous self-assembly of PLPC into a bilayer. The PLPC and arbutin is depicted as yellow and cyan respectively, blue is nitrogen atom in phospholipids, red is oxygen atom in conjugates. Note that water molecules are not represented.


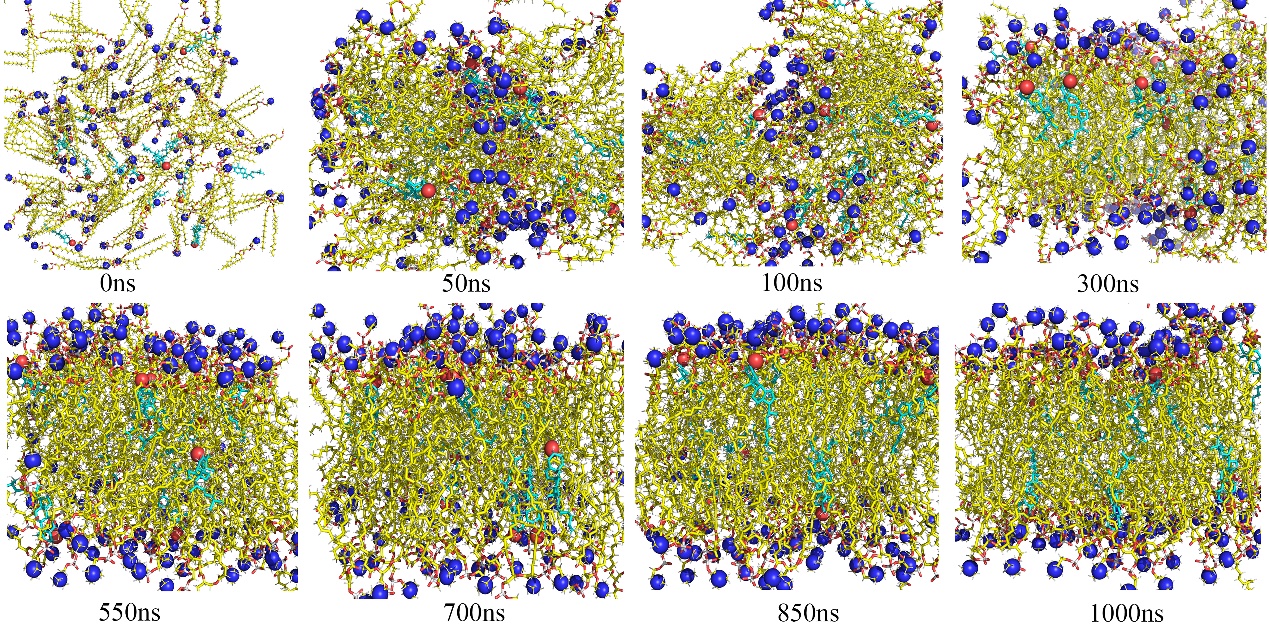


**Figure S4**: Snapshots showing the spontaneous self-assembly of PLPC into a bilayer. The PLPC and cholesterol is depicted as yellow and cyan respectively, blue is nitrogen atom in phospholipids, red is oxygen atom in conjugates. Note that water molecules are not represented.


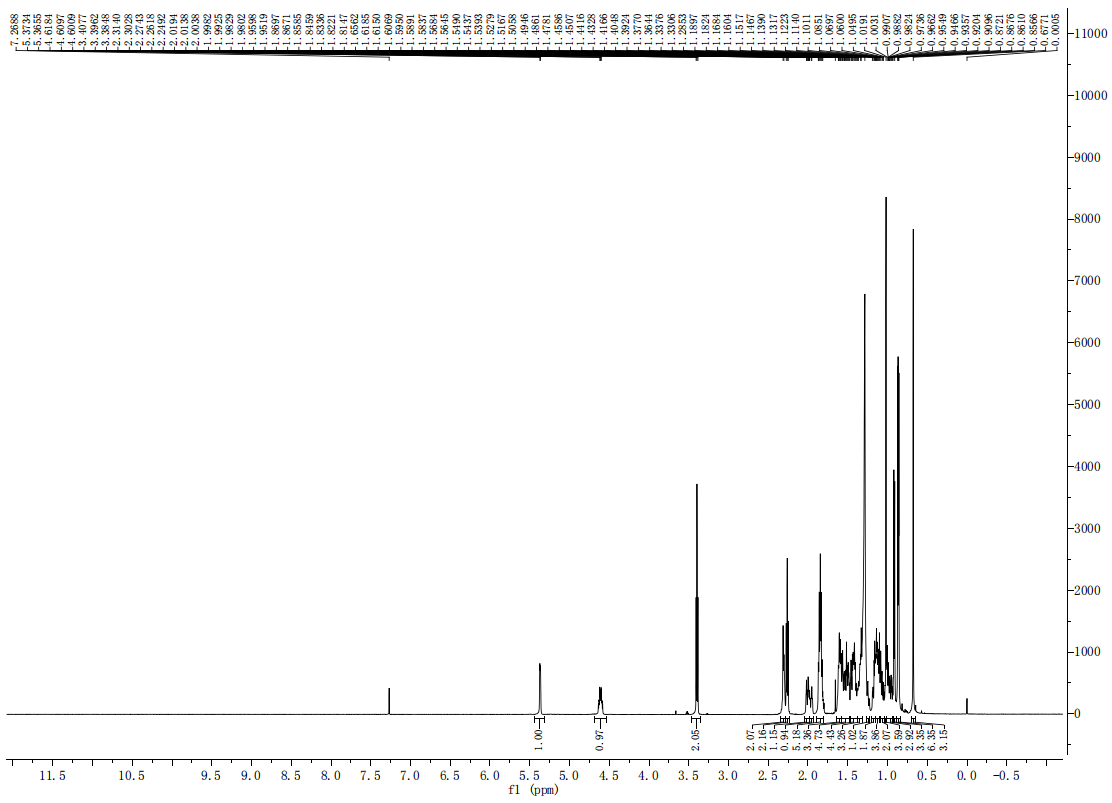


**Figure S5** ^1^H NMR spectrum of 3-(11-bromoundecanoate)-cholesterol.


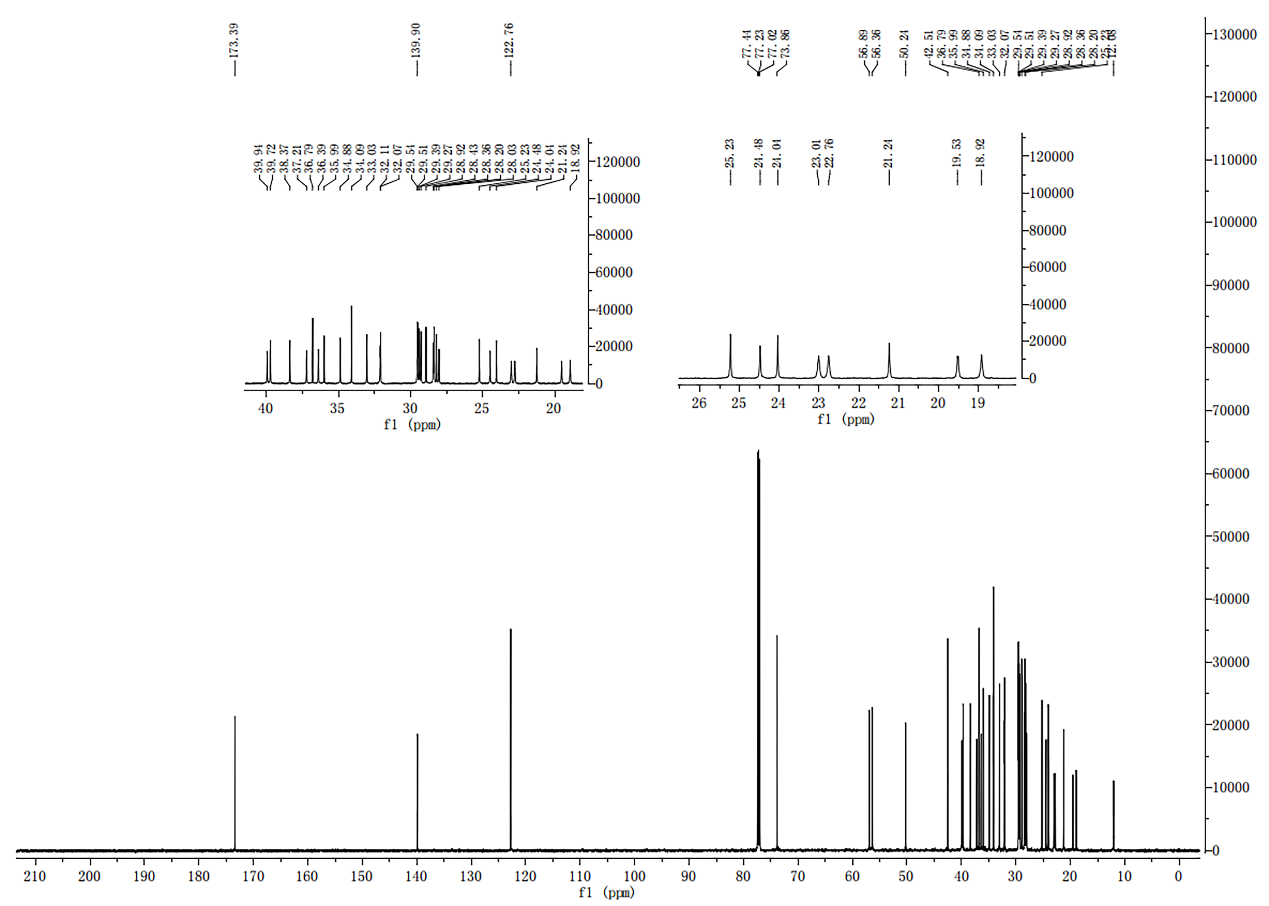


**Figure S6** ^13^C NMR spectrum of 3-(11-bromoundecanoate)-cholesterol.

**
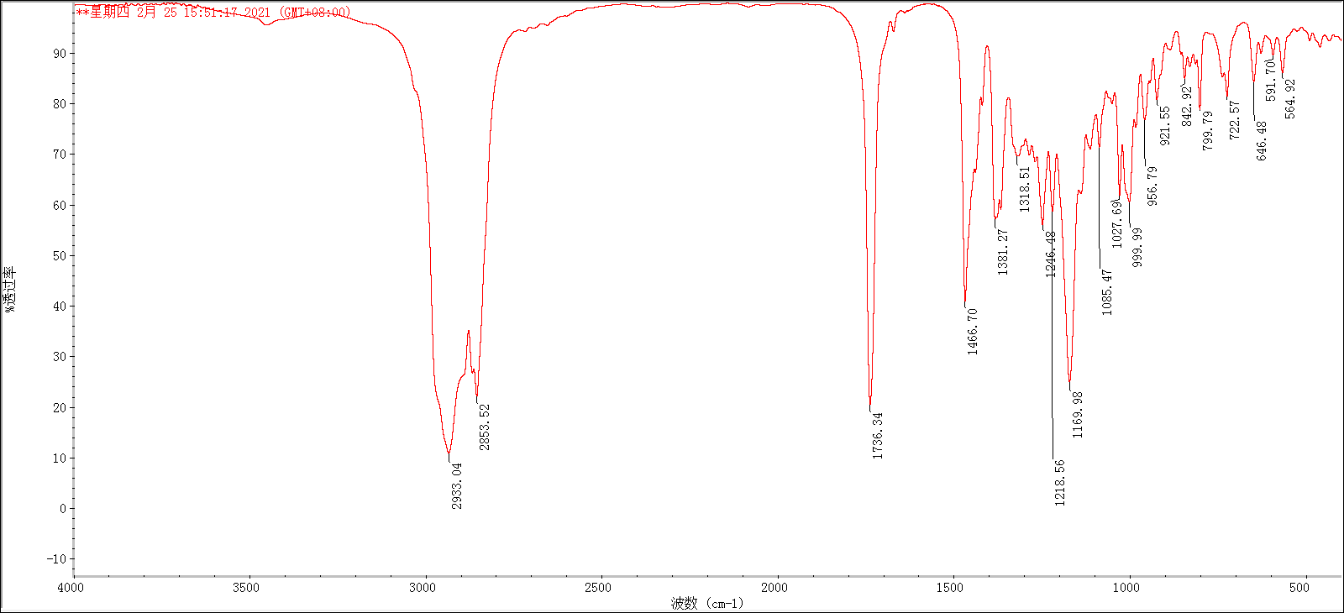
**

**Figure S7** Infrared spectrum of 3-(11-bromoundecanoate)-cholesterol.

**
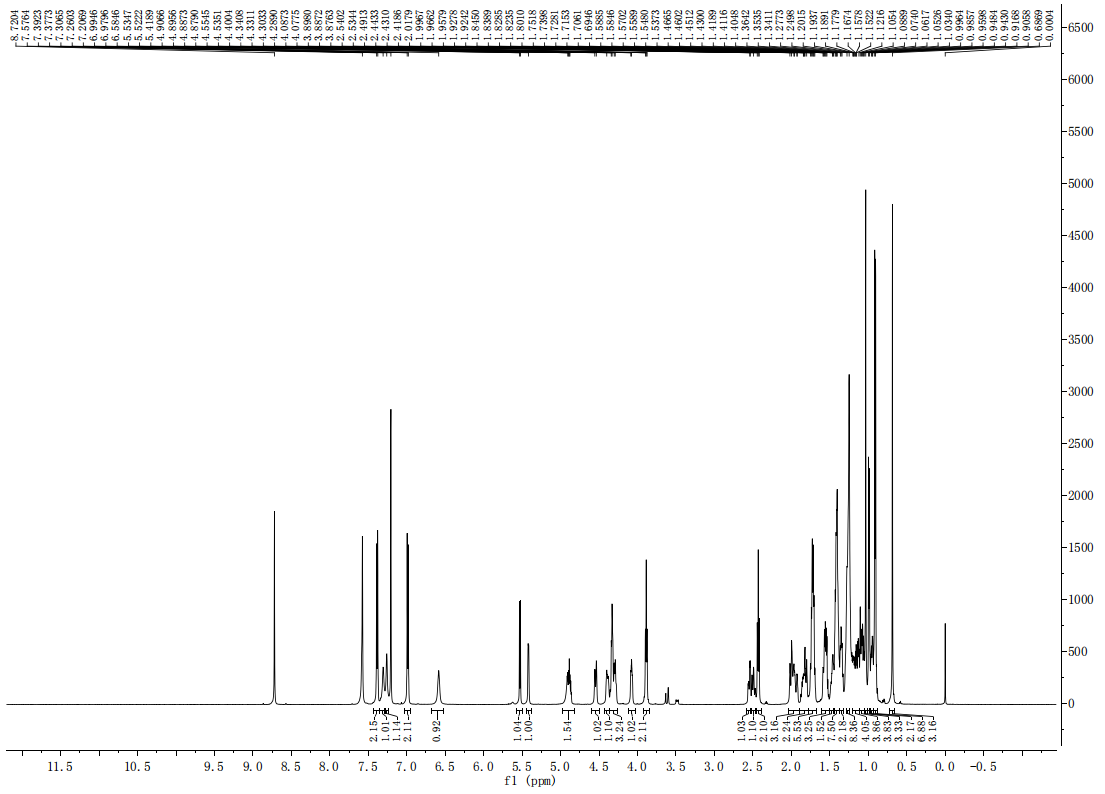
**

**Figure S8** ^1^H NMR spectrum of cholesterol-undecanoate-glucose conjugate.


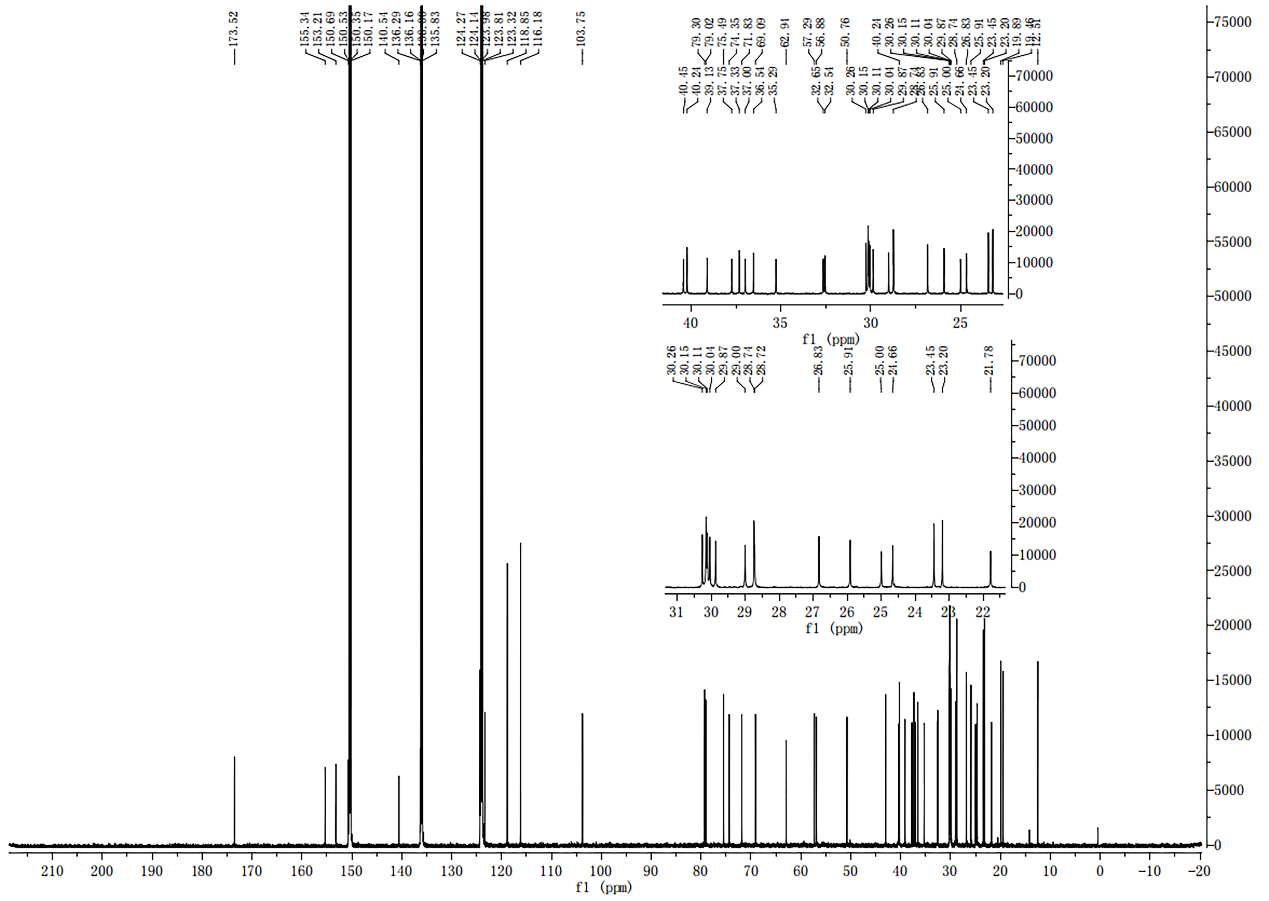


**Figure S9** ^13^C NMR spectrum of cholesterol-undecanoate-glucose conjugate.

**
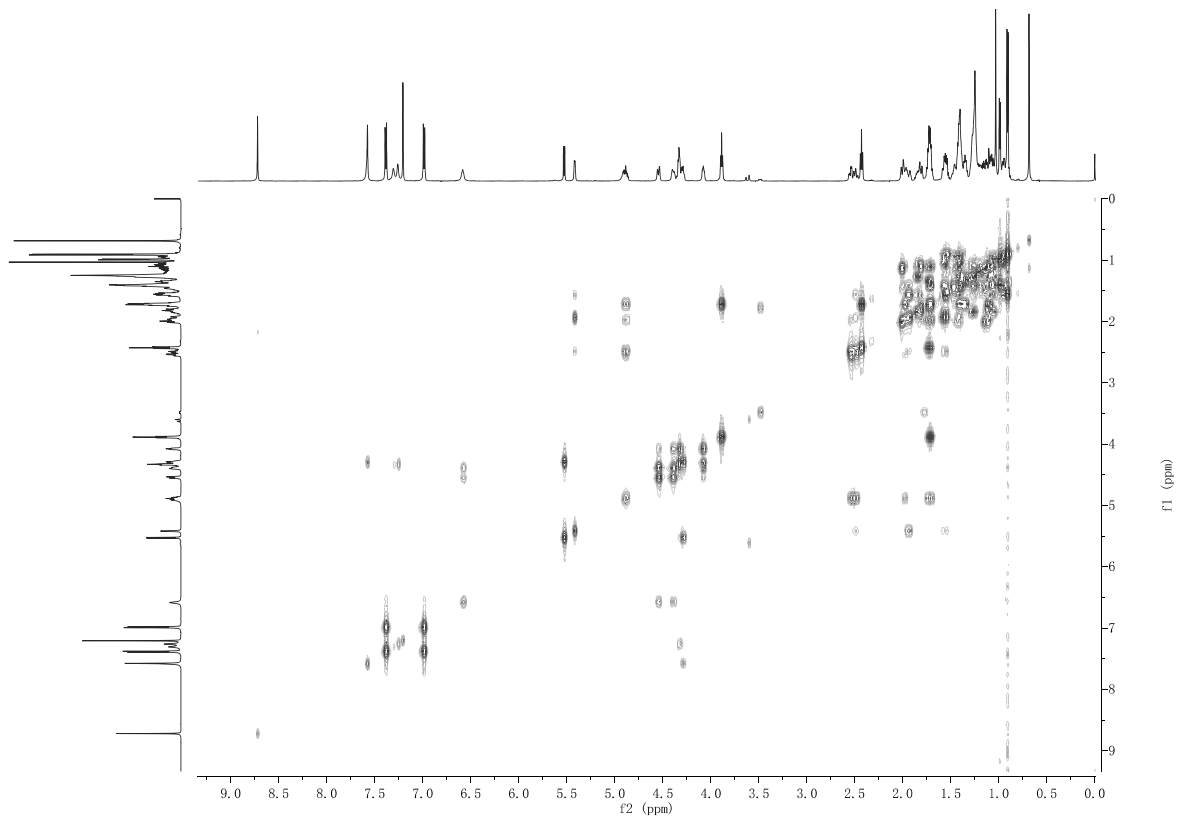
**

**Figure S10** COSY spectrum of cholesterol-undecanoate-glucose conjugate.

**
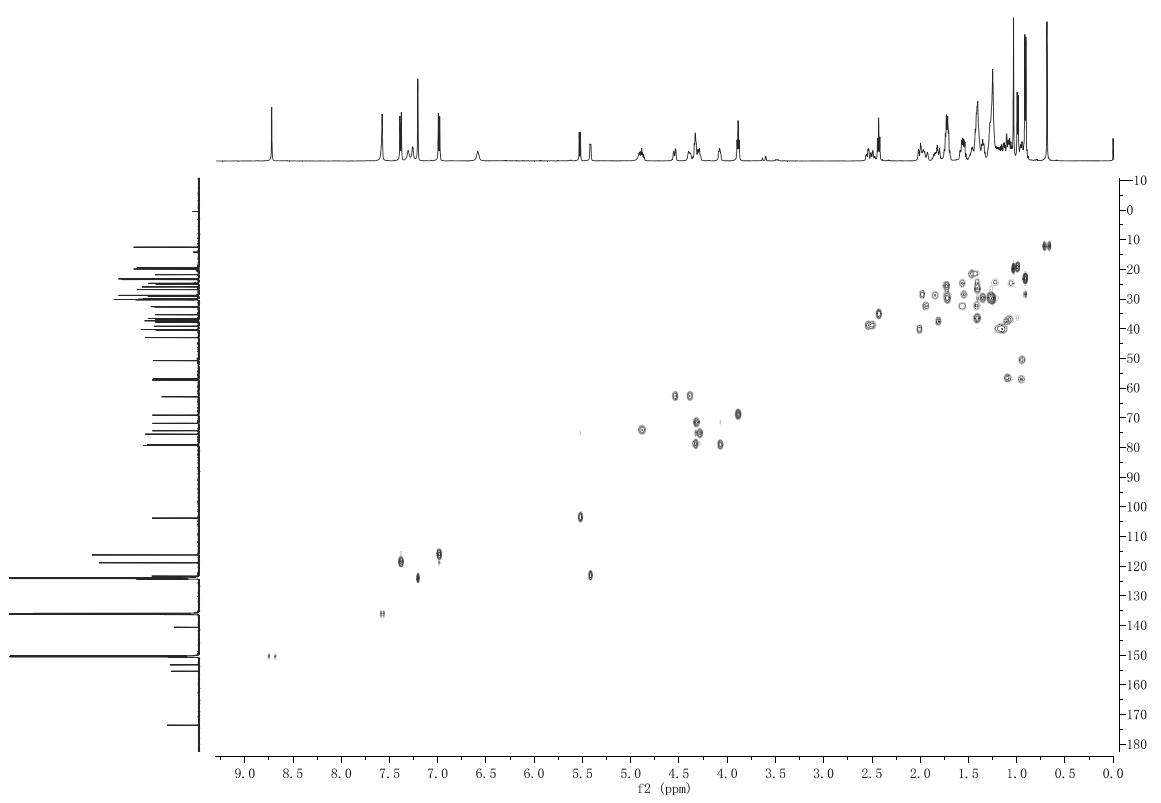
**

**Figure S11** HSQC spectrum of cholesterol-undecanoate-glucose conjugate.

**
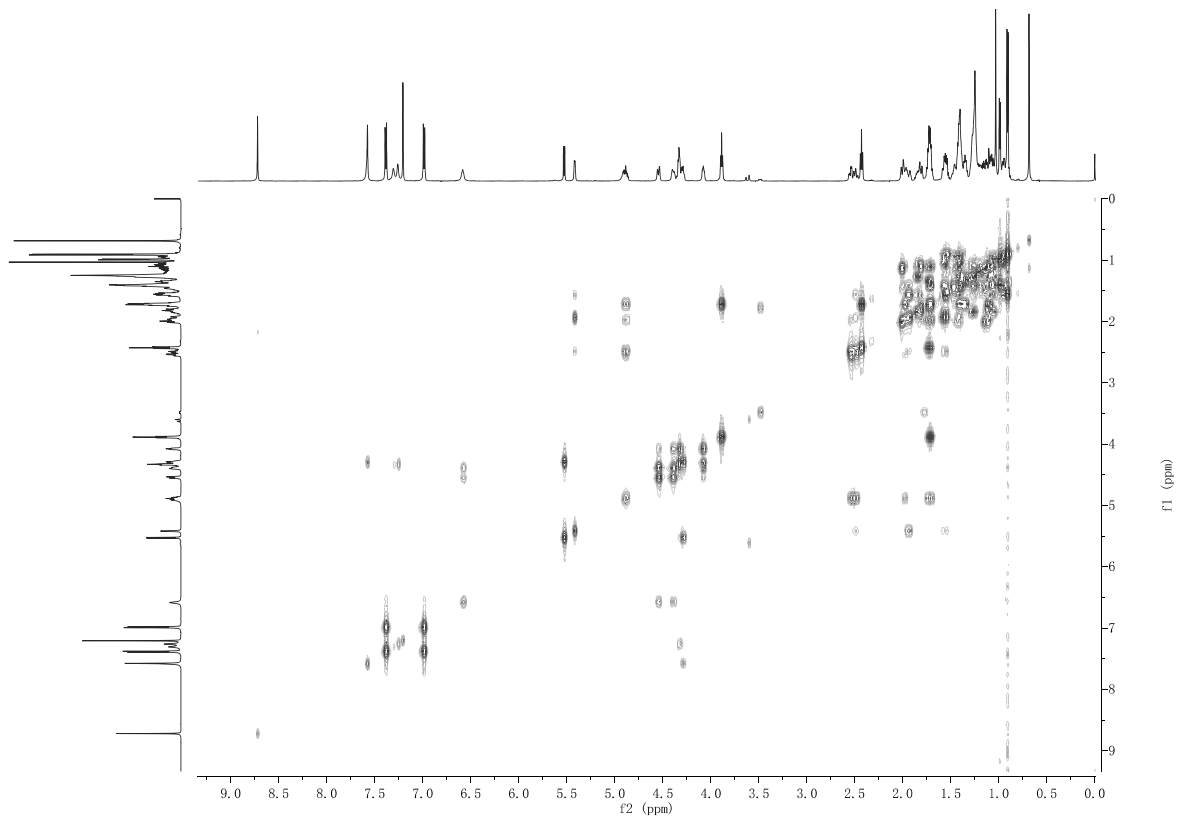
**

**Figure S12** HMBC spectrum of cholesterol-undecanoate-glucose conjugate.

**
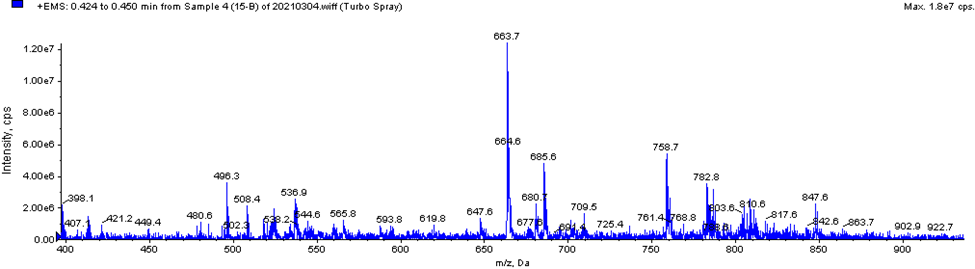
**

**Figure S13** ESI mass spectrum of cholesterol-undecanoate-glucose conjugate.

**
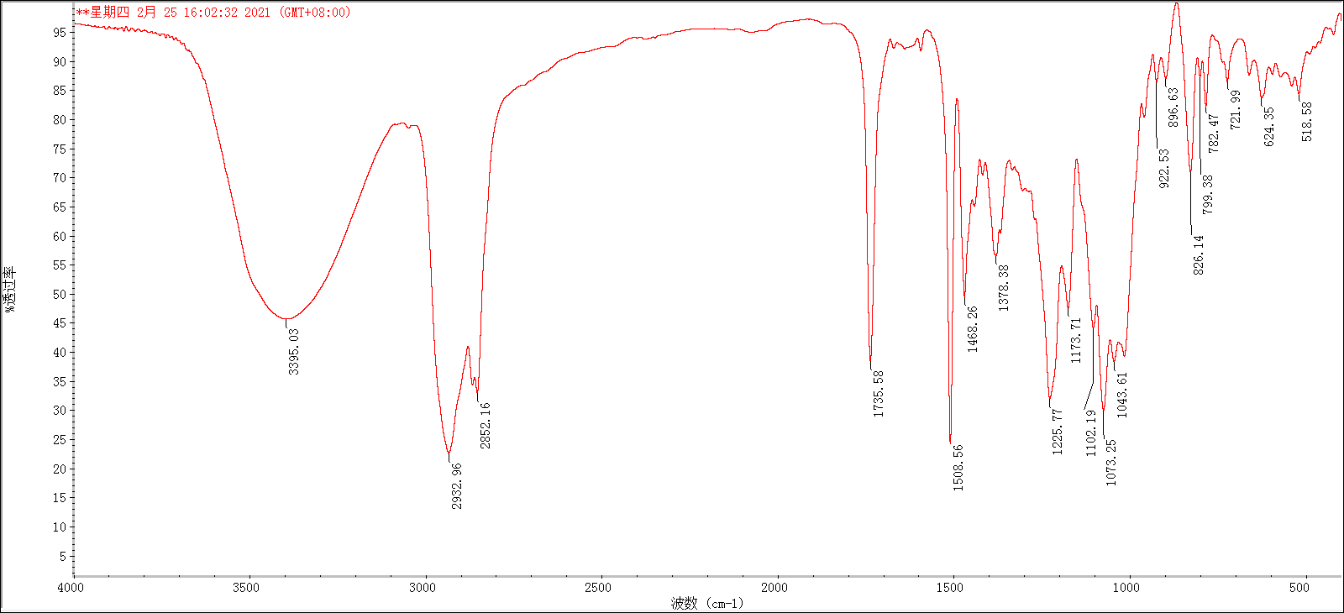
**

**Figure S14** Infrared spectrum of cholesterol-undecanoate-glucose conjugate.


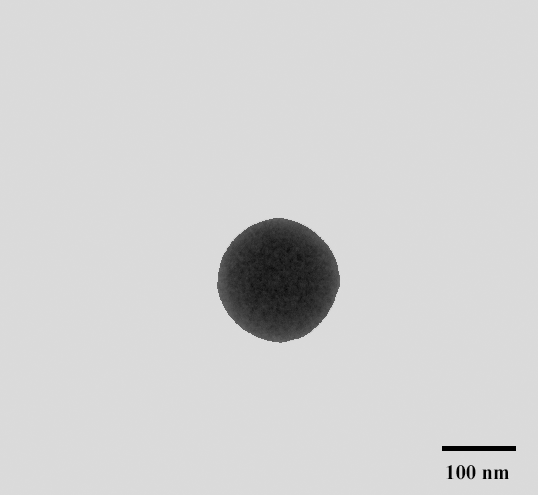


**Figure S15** TEM image of iv-ART/TMP@lipoBX.


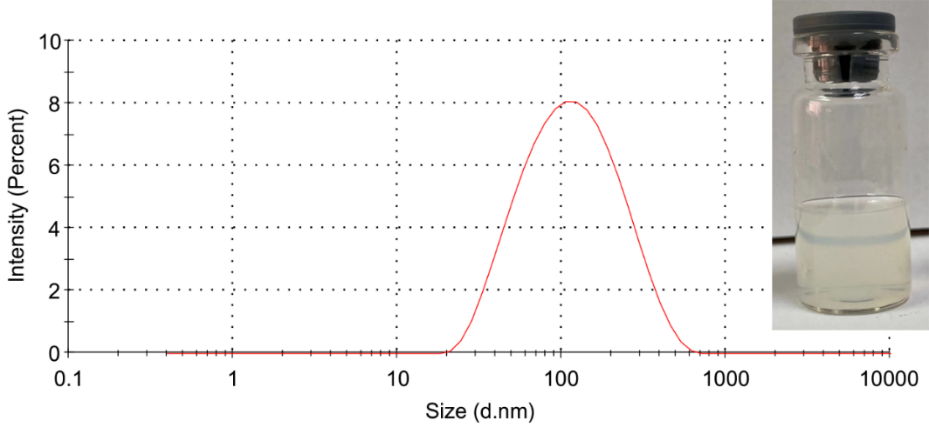


**Figure S16** Particle size distribution of iv-ART/TMP@lipoBX.





**Figure S17** DSC spectrum of iv-ART/TMP@lipoBX.
